# POLRMT overexpression increases mtDNA transcription without affecting steady-state mRNA levels

**DOI:** 10.26508/lsa.202302563

**Published:** 2025-10-17

**Authors:** Maria Miranda, Andrea Mesaros, Nathalie Scrima, Louise Pérard, Irina Kuznetsova, Martin Purrio, Ilian Atanassov, Aleksandra Filipovska, Arnaud Mourier, Nils-Göran Larsson, Inge Kühl

**Affiliations:** 1 Max Planck Institute for Biology of Ageing , Cologne, Germany; 2 Université Paris-Saclay, CEA, CNRS, Institute for Integrative Biology of the Cell, Gif-sur-Yvette, France; 3 The Kids Research Institute Australia, Northern Entrance, Perth Children’s Hospital, Nedlands, Australia; 4 ARC Centre of Excellence in Synthetic Biology, University of Western Australia, Crawley, Australia; 5 Université de Bordeaux, CNRS, IBGC, UMR 5095, Bordeaux, France; 6 Department of Medical Biochemistry and Biophysics, Karolinska Institutet , Stockholm, Sweden

## Abstract

POLRMT is the sole RNA polymerase in human mitochondria, where it generates primers for mitochondrial DNA (mtDNA) replication and transcribes the mtDNA to express genes encoding essential components of the oxidative phosphorylation (OXPHOS) system. Elevated POLRMT levels are found in several cancers and in mouse models with severe mitochondrial dysfunction. Here, we generated and characterized mice overexpressing *Polrmt* to investigate the physiological and molecular consequences of elevated POLRMT levels. Increasing POLRMT levels did not result in any pathological phenotype but led to increased exercise performance in male mice under stress conditions. *Polrmt* overexpression increased mtDNA transcription initiation, resulting in higher steady-state levels of the promoter-proximal L-strand transcript *7S* RNA. Surprisingly, the abundance of mature mitochondrial RNAs was not affected by the elevated POLRMT levels. Furthermore, ubiquitous simultaneous overexpression of *Polrmt* and *Lrpprc*, which stabilizes mitochondrial messenger RNAs, did not increase steady-state levels of mitochondrial transcripts in the mouse. Our data show that POLRMT levels regulate transcription initiation, but additional regulatory steps downstream of transcription initiation and transcript stability limit OXPHOS biogenesis.

## Abstract (precis)

This study investigates effects of increased POLRMT levels and reports stimulation of mtDNA transcription initiation and enhanced exercise capacity without an effect on steady-state mRNA levels.

## Introduction

Biogenesis of the mitochondrial oxidative phosphorylation (OXPHOS) system is vital for mammalian life as it fulfils several metabolic functions, including the generation of most cellular energy in the form of ATP, and altered OXPHOS capacity is implicated in a broad range of human pathologies, including cancer and aging (Larsson, 2010; Whitehall & Greaves, 2020). The OXPHOS system is under dual genetic control by the nuclear and mitochondrial genomes, i.e., nDNA and mtDNA, respectively (Falkenberg et al, 2024). In mammals, the nDNA encodes most of the ∼1,200 mitochondrial proteins, including all factors required to express and maintain mtDNA and the vast majority of the ∼90 OXPHOS subunits (Morgenstern et al, 2021; Rath et al, 2021). In contrast, mammalian mtDNA only encodes 13 proteins that are all essential subunits of four of the five OXPHOS complexes, as well as two mitochondrial ribosomal RNAs (mt-rRNAs) and 22 transfer RNAs (mt-tRNAs) needed for mitochondrial protein synthesis. Failure to express mtDNA causes a global loss of mitochondrial protein complexes with dual genetic contributions, i.e., the OXPHOS complexes and the mitochondrial ribosome (Kühl et al, 2017), suggesting that regulation of mtDNA gene expression is not only essential for mitochondrial function but also allows local and rapid adaptations to bioenergetic and metabolic demands. Mammalian mtDNA is an intron-free, circular molecule present in hundreds to thousands of copies in most mammalian cell types. The mammalian mtDNA is not naked but fully coated and condensed into mitochondrial nucleoids by mitochondrial transcription factor A (TFAM) and other proteins (Kukat et al, 2015; García-Villegas et al, 2025). The compaction of the nucleoid varies; and the more relaxed form is accessible for mtDNA replication and transcription, whereas the more condensed form may serve to protect and store the genome (Bonekamp et al, 2021; Isaac et al, 2024; García-Villegas et al, 2025). The mammalian mtDNA is composed of two coding strands known as the heavy (H) and the light (L) strand because of the differences in their G+T base composition (Falkenberg et al, 2024). Mammalian mtDNA is only ∼16.5 kb in size, is densely packed with genes, and has only one long noncoding control region (NCR) of ∼1 kb. The NCR contains the H-strand origin of replication (O_H_) and promoters for transcription initiation of the H strand (HSP) and L strand (LSP). A second LSP was reported in human and apes (Tan et al, 2022; Falkenberg et al, 2024). Transcription of mtDNA generates near genome-length polycistronic transcripts that are processed to yield individual, mature RNA molecules (mt-RNAs) (Ojala et al, 1981; Montoya et al, 1982; Holzmann et al, 2008; Rackham et al, 2016; Siira et al, 2018). The link between posttranscriptional RNA processing and translation is not fully understood, but it has been shown that the leucine-rich pentatricopeptide repeat–containing protein (LRPPRC) and the stem–loop interacting RNA binding protein (SLIRP) form a heterodimeric protein complex that promotes polyadenylation and stabilizes all mt-mRNAs except the L-strand encoded *mt-Nd6* (Gohil et al, 2010; Sasarman et al, 2010; Ruzzenente et al, 2012; Harmel et al, 2013; Rubalcava-Gracia et al, 2024).

The basal machinery for mitochondrial transcription is well understood and only consists of a limited set of proteins that are encoded by the nDNA and imported into mitochondria (Miranda et al, 2022; Falkenberg et al, 2024). The single-subunit mitochondrial RNA polymerase (POLRMT) operates exclusively in mitochondria (Ringel et al, 2011; Kühl et al, 2014, 2016) where it interacts with TFAM and mitochondrial transcription factor B2 (TFB2M) to initiate transcription (Hillen et al, 2017; Falkenberg et al, 2024). After initiation, TFAM and TFB2M leave POLRMT, which instead binds the mitochondrial transcription elongation factor (TEFM) to allow polycistronic transcription of both strands (Minczuk et al, 2011; Jiang et al, 2019). Transcription termination of the L-strand depends on the mitochondrial transcription termination factor 1 (MTERF1), whereas factors involved in termination of H-strand transcription remain to be clarified (Terzioglu et al, 2013). It should be emphasized that data from in vitro reconstituted pure recombinant systems, as well as atomic structures of transcription protein complexes (Wanrooij et al, 2008; Schwinghammer et al, 2013; Morozov et al, 2015; Posse et al, 2015; Hillen et al, 2018; Herbine et al, 2025) and several conditional knockout mouse models (Larsson et al, 1998; Kühl et al, 2016; Matic et al, 2018; Jiang et al, 2019), support the current models for transcription initiation, elongation, and termination (Miranda et al, 2022; Falkenberg et al, 2024). Despite this considerable progress in elucidating the basal molecular machinery for mtDNA transcription, the regulatory mechanisms remain poorly understood. Although several studies have suggested intramitochondrial roles for some nuclear transcription factors, this area remains controversial because biochemical in vitro reconstitution experiments and structural studies of how these nuclear factors directly interact with the basal mitochondrial transcription machinery are lacking (Rubalcava-Gracia et al, 2023).

Importantly, POLRMT also generates the RNA primers required for mtDNA replication initiation at the L-strand origin of replication (O_L_) and O_H_ (Kühl et al, 2016; Falkenberg, 2018; Sarfallah et al, 2021), placing this enzyme at the core of both mtDNA expression and maintenance. A proportion of LSP transcripts is terminated in the NCR (Wanrooij et al, 2012) to generate RNA primers for DNA synthesis at O_H_ (Falkenberg et al, 2024). The regulation of transcription for primer formation versus gene expression is partly understood, and different factors such as mitochondrial RNase H1 and mitochondrial single-strand binding protein (SSBP1) are of key importance in this process (Agaronyan et al, 2015; Kühl et al, 2016; Jiang et al, 2021; Misic et al, 2022).

Recent studies have highlighted the medical importance of understanding the regulation of mitochondrial transcription. Pathogenic mutations in *POLRMT* cause mitochondrial dysfunction with a broad spectrum of neurodevelopmental presentations in humans because of defective mitochondrial transcription (Oláhová et al, 2021). Elevated POLRMT levels are found in patients with lung and breast cancer (Sotgia et al, 2012) as well as in acute myeloid leukemia cells (Bralha et al, 2015; Chaudhary et al, 2021) and prostate cancer (Li et al, 2023). This association of elevated PORLMT levels with some cancers has led to the suggestion that *POLRMT* may be a novel important oncogene (Zhou et al, 2021; Miranda et al, 2022). Consistent with its putative role in cancer, POLRMT has been proposed as a therapeutic target to treat some cancers and specific small molecule inhibitors have been developed (Bonekamp et al, 2020; Li et al, 2023; Wang et al, 2024). Furthermore, increased POLRMT levels are found in many different mouse models with mitochondrial dysfunction (Milenkovic et al, 2013; Kühl et al, 2017; Jiang et al, 2019; Silva Ramos et al, 2019). It is unknown whether the increased POLRMT levels should be regarded as part of a compensatory mitochondrial biogenesis response or if they contribute to pathogenesis by worsening mitochondrial disease phenotypes. Here, we investigated the physiological and molecular consequences of elevated POLRMT levels in nonpathogenic conditions by generating and characterizing mice ubiquitously overexpressing *Polrmt*. The *Polrmt*-overexpressing mice are viable and have no evident pathologic phenotype by 1 yr of age, the latest time point studied. Remarkably, overexpression of *Polrmt* had a positive effect on performance under exercise challenge conditions. Molecular analysis of various tissues of the *Polrmt*-overexpressing mice suggests that POLRMT is limiting for mtDNA transcription initiation because elevated POLRMT levels increase de novo transcription and steady-state levels of the promoter-proximal *7S* RNA transcript of the L-strand. Under normal conditions, this increase in mtDNA gene expression is not translated into an increase in OXPHOS capacity because mature mitochondrial transcripts are not globally up-regulated. Our findings support that regulatory checkpoints occur downstream of transcription initiation, balancing mtDNA transcription elongation and posttranscriptional processes depending on energetic needs in vivo.

## Results

### Overexpression of *Polrmt* results in healthy mice with normal OXPHOS

**Figure 1.**
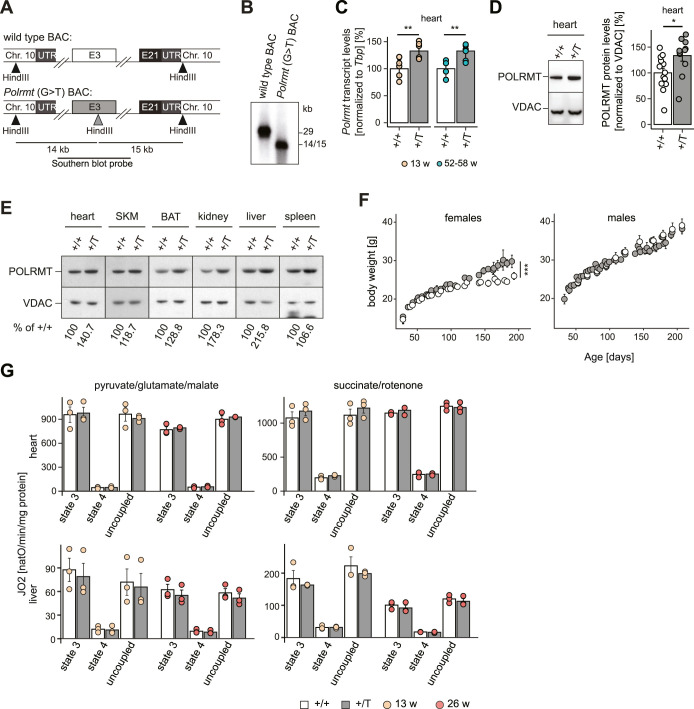
*Polrmt*-overexpressing mice are viable. **(A)** Scheme of BAC modification strategy. Chr, chromosome; E, exons; grey triangle indicates introduced HindIII restriction site and black triangles indicate endogenous restriction sites. Size of the DNA fragments generated after HindIII restriction and location of Southern blot are indicated at the bottom. **(B)** Representative Southern blot of BAC construct after HindIII restriction digest. **(C)** qRT-PCR analysis of steady-state *Polrmt* transcript levels in WT (+/+) and *Polrmt* overexpressor (+/T) mouse hearts at different ages. Normalization: TATA-binding protein (*Tbp*); n: 4–6 per genotype per age. **(D, E)** Representative Western blot of POLRMT levels in mitochondrial extracts +/+ and +/T from the heart with quantification (n: 11–13) (D) and from different tissues of a 52-wk-old mouse (n: 1) (E). Loading: VDAC; SKM, skeletal muscle; BAT, brown adipose tissue. **(F)** Body weight curves of female (upper) and male mice (lower); n: 7–16 per sex and genotype. g, grams; grey:+/T, white:+/+; ***P < 0.001 ANOVA repeated measurements **(G)** Oxygen consumption analysis on isolated mitochondria from the heart and liver. Mitochondria were incubated with pyruvate, glutamate, and malate to deliver electrons to complex I or with succinate and rotenone to deliver electrons to complex II (CII). Mitochondrial respiration was analyzed under phosphorylating (state 3), non-phosphorylating (state 4), and uncoupled conditions. n: 3 per age. Percentage (%) is calculated relative to +/+ levels. Error bars ± SEM. *P < 0.05, ***P < 0.001; +/T and +/+ comparisons at different ages were tested within each age using a linear model with Tukey-adjusted pairwise tests. Source data are available for this figure.

**Figure S1.**
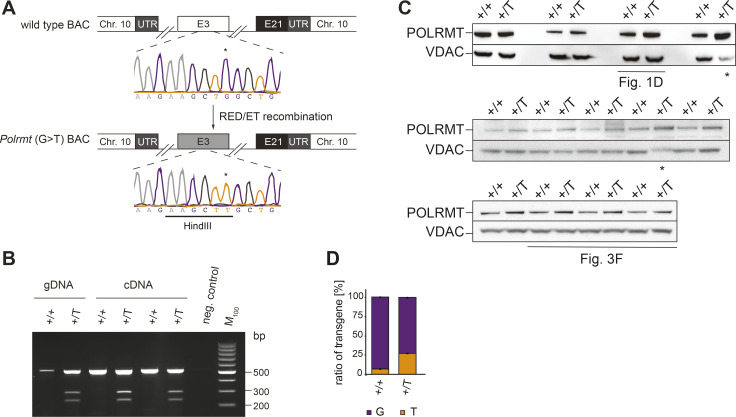
Generation of endogenous *Polrmt*-overexpressing mice. **(A)** Detailed scheme of BAC modification strategy. Chr, chromosome; E, exons; asterisk point mutation introduced in c420. Sequencing chromatogram is shown. **(B)** PCR and restriction digest analysis of the *Polrmt* (G>T) BAC transgenic allele in genomic (gDNA) and reverse transcribed RNA (cDNA) from WT (+/+) and BAC transgenic *Polrmt* overexpressor (+/T) mice. **(C)** Western blots of POLRMT levels in mitochondrial extracts +/+ and +/T from the heart quantified in Fig 1D. 2 samples (*) were excluded from quantification because of issues with VDAC. **(D)** Pyrosequencing analysis of the ratio of WT (G) and transgenic (T) alleles in DNA isolated from tail biopsies in +/+ and +/T mice; n: 9 per genotype and sex.

**Figure S2.**
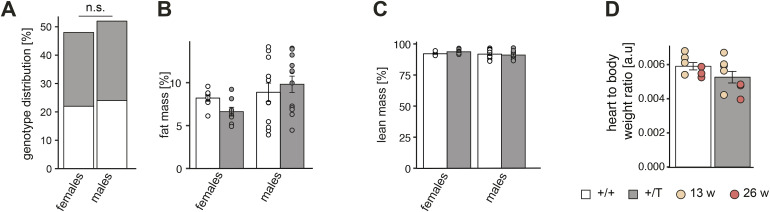
*Polrmt*-overexpressing mice are healthy. **(A)** Histogram of genotype distribution of the WT (+/+, white) and *Polrmt*-overexpressing (+/T, grey) offspring; n = 22–28 per genotype per sex, n.s: *P* > 0.05; chi-square test. **(B, C)** Fat mass percentage and (C) Lean mass percentage of 10-wk-old +/+ and +/T mice before starting the exercise challenge; n: 8–12 per sex per genotype. **(D)** Heart-to-body weight ratio at different ages; n: 3–5 per age and genotype.

**Figure S3.**
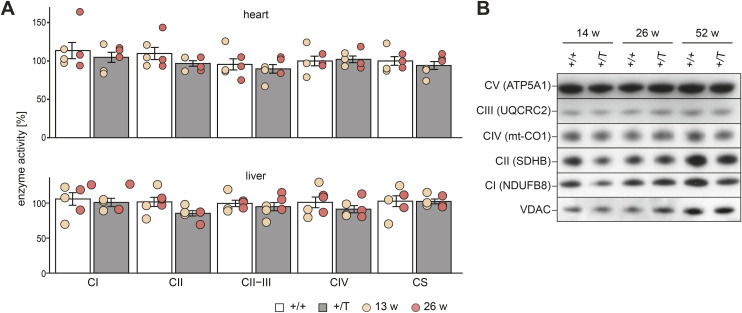
Bioenergetic analyses in the heart and liver mitochondria. **(A)** Relative enzyme activity of respiratory chain enzymes measured in mitochondria isolated from the heart and liver at different ages. The enzymes measured are: Complex I: NADH ubiquinone oxidoreductase, Complex II: succinate dehydrogenase, Complex II-III: Succinate dehydrogenase—cytochrome c reductase, Complex IV: Cytochrome c oxidase, and citrate synthase (CS). n: 3 per age. **(B)** Western blot of OXPHOS subunit levels in isolated mitochondria from the heart at different ages; loading: VDAC. n: 3. Error bars ± SEM.

To study the role of moderately increased POLRMT levels, we generated mice ubiquitously overexpressing *Polrmt* using a bacterial artificial chromosome (BAC) transgenic strategy (Park et al, 2007). A BAC clone containing a fragment of mouse chromosome 10 with the *Polrmt* gene was modified to introduce a silent point mutation (c.420G>T) that generates a HindIII restriction site in exon 3, thus differentiating the introduced transgene from the endogenous *Polrmt* alleles (Figs 1A and B and S1A). This BAC transgenic strategy allows an increased expression of *Polrmt* regulated by its endogenous promotor and is predicted to increase *Polrmt* expression within a relevant physiological range. Germline transmission and expression of the transgene was verified by PCR and subsequent HindIII restriction digestion (Fig S1B). We observed ∼33% increase in *Polrmt* transcript levels (Fig 1C) as well as a comparable increase in POLRMT protein levels in the heart (Figs 1D and S1C). POLRMT levels were also increased in various other tissues such as the brown adipose tissue, kidney, liver, and spleen (Fig 1E). Quantification of the transgene copy number by pyrosequencing confirmed that a single copy was integrated into the mouse genome (Fig S1D). We did not obtain founders containing more than one additional copy of the *Polrmt* gene. The *Polrmt*-overexpressing mice were born at Mendelian ratios (Fig S2A) and had a normal body weight gain with females showing a slight but significant increase in body weight from 150 d of age (Fig 1F). Proportions of fat and lean mass were normal (Fig S2B and C), and *Polrmt*-overexpressing mice showed no abnormal behavior up until 52 wk of age. Furthermore, there was no evidence of cardiomyopathy as the heart-to-body weight ratios were normal at 13 and 26 wk (Fig S2D). No mice were euthanized because of abnormal cancerous masses during the course of the study. We next proceeded to evaluate whether moderately increased levels of POLRMT influenced the mitochondrial bioenergetic capacity. We found normal respiration of isolated mitochondria from the heart and liver incubated with complex I or complex II substrates and analyzed under phosphorylating (state 3), non-phosphorylating (state 4), or uncoupled conditions (Fig 1G), indicating normal OXPHOS function. To further verify our findings, we performed enzyme activity assays and Western blot analyses for respiratory chain complexes and found no differences between wild-type (WT) and *Polrmt*-overexpressing mice (Fig S3A and B). These results show that a moderate increase in POLRMT levels does not affect OXPHOS function or cause pathology.

### Moderately elevated POLRMT levels have a positive effect on exercise capacity

**Figure S4.**
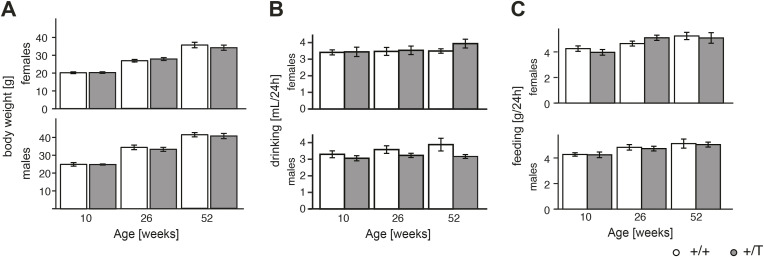
Phenomaster parameters. **(A)** Body weight of female and male WT (+/+) and *Polrmt*-overexpressing (+/T) mice at different ages at the beginning of the indirect calorimetry experiment. **(B)** Water consumption. **(C)** Food consumption. n: 8–9 mice/genotype.

**Figure 2.**
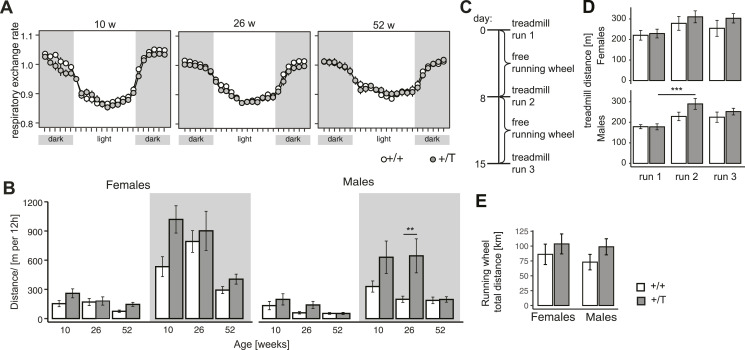
*Polrmt*-overexpressing mice have increased exercise capacity. **(A)** Respiratory exchange rate normalized to lean body mass in female and male +/+ and +/T mice at different ages measured in metabolic cages; ticks in x-axis are spaced by 1 h; grey background, dark cycle; white background, light cycle; n: 7–9 mice/genotype/sex. **(B)** Cumulative distance travelled per day in +/+ and +/T mice at different ages; grey background, dark cycle; white background, light cycle; n: 7–9 mice/genotype/sex. **(C)** Scheme exercise challenge on 10–12-wk-old females and males +/+ and +/T mice. **(D)** Distance run on treadmill challenge at day 0, 8, and 15. n: 8–12 mice/genotype/sex. **(E)** Distance run in free running wheels during the exercise challenge. n: 8–12 mice/genotype/sex. Error bars ± SEM. *P < 0.05, ***P < 0.001, ANOVA. +/T and +/+ comparisons at different ages were tested within each age using a linear model, adjusted for repeated measurements, and with Tukey-adjusted pairwise tests. Source data are available for this figure.

We assessed the effect of elevated POLRMT levels on animal physiology at 10, 26, and 52 wk of age. Using metabolic cages, we monitored the energy homeostasis and activity of *Polrmt*-overexpressing mice and WT littermates during the light and dark cycle. We did not detect significant differences in the drinking and feeding behavior or body weight gain (Fig S4A–C), showing that the overexpressing mice are stress free when kept under standard housing conditions. We determined the respiratory exchange ratio by measuring the O_2_ consumption and CO_2_ production and found no differences in substrate utilization in vivo between WT and *Polrmt*-overexpressing mice (Fig 2A). Surprisingly, when we studied the cumulative distance traveled in the metabolic chambers, we observed that *Polrmt*-overexpressing mice tended to display increased activity compared with control mice (Fig 2B). This increase was significant in males at 26 wk of age in the dark cycle, when mice are typically more active. We proceeded to study whether elevated POLRMT levels could be beneficial for exercise performance. Using monitored free-running wheels and several treadmill runs, we challenged the *Polrmt*-overexpressing and littermate control mice of both sexes to a voluntary and strenuous exercise regime (Fig 2C). Interestingly, voluntary exercise on free running wheels in the *Polrmt*-overexpressing mice significantly increased the capacity on a treadmill in comparison with controls in male mice (Fig 2D and E). The increased distance run on the treadmill test at 10–12 wk of age and the increased activity in 26-wk-old male mice show that moderate *Polrmt* overexpression can increase exercise capacity, the maximum amount of physical exertion that the mice can sustain (Goldstein, 1990).

### *Polrmt* overexpression has no effect on mtDNA levels

**Figure 3.**
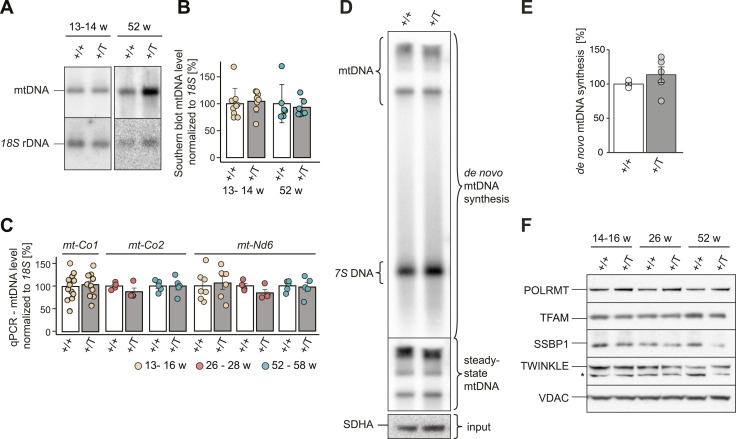
*Polrmt* overexpression leads to unaltered mtDNA copy number. **(A)** Representative Southern blot analysis of mtDNA levels at different ages. **(B)** Quantification of mtDNA levels from Southern blot at different ages in WT (+/+, white) and *Polrmt* overexpressor (+/T, grey) mice. Normalization *18S* rDNA.; n: 4–6. **(C)** Quantification of mtDNA levels by qPCR using specific probes against *mt-Co1*, *mt-Co2*, *mt-Nd6*, and *18S* rDNA. n > 4 per genotype. +/T and +/+ comparisons at different ages were tested within each age using a linear model. **(D, E)** Representative in organello replication on isolated mitochondria from the heart and (E) quantification of three experiments. Input: Western blot of SHDA and steady-state mtDNA levels. Normalization: protein input; n: 5 per genotype. Error bars ± SEM; grey:+/T, white:+/+. **(F)** Western blot of factors required for mtDNA replication expression in +/+ and +/T mice. Loading: VDAC; n: 3–4 per genotype; asterisk: unspecific band (Kühl et al, 2016). Source data are available for this figure.

**Figure S5.**
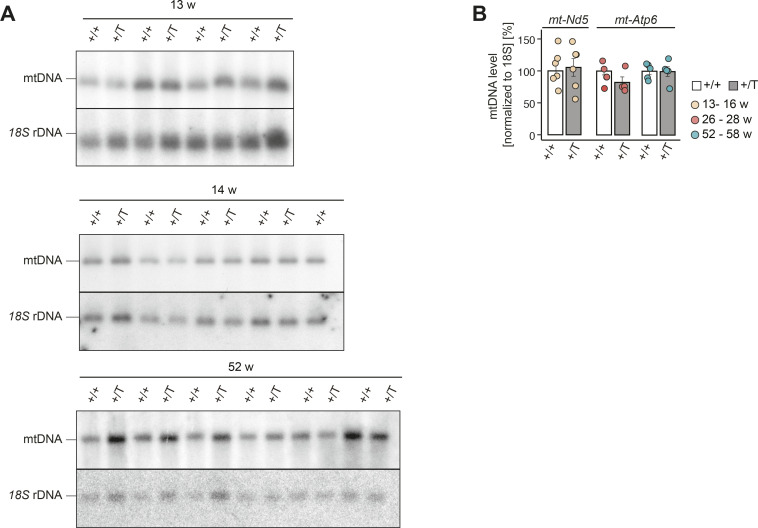
mtDNA quantifications. **(A)** Images of Southern blot experiments quantified in Fig 3B. **(B)** Quantification of mtDNA levels by qPCR using specific probes against *mt-Nd5*, *mt-Atp6*, and *18S* rDNA. n > 4 per genotype. +/T and +/+ comparisons at different ages were tested within each age using a linear model.

Given the essential role of POLRMT to serve as the primase that generates RNA primers for mtDNA replication (Falkenberg et al, 2024), we evaluated whether *Polrmt* overexpression affected mtDNA replication. We determined the steady-state mtDNA copy number by Southern blot and qPCR and found no significant changes in mtDNA levels in the heart of *Polrmt*-overexpressing mice at 14, 26, and 52 wk of age (Figs 3A–C and S5A and B). In organello mtDNA synthesis in isolated mitochondria showed a consistent trend toward an increase, but this difference was not statistically significant (Fig 3D and E). We further determined steady-state levels of essential factors for these processes but found no changes in *Polrmt*-overexpressing mice (Fig 3F). Our data show that a moderate elevation of POLRMT levels has no effect on mtDNA levels or TFAM levels (Figs 3F and S5A and B).

### POLRMT levels may limit mitochondrial transcription in vivo

**Figure 4.**
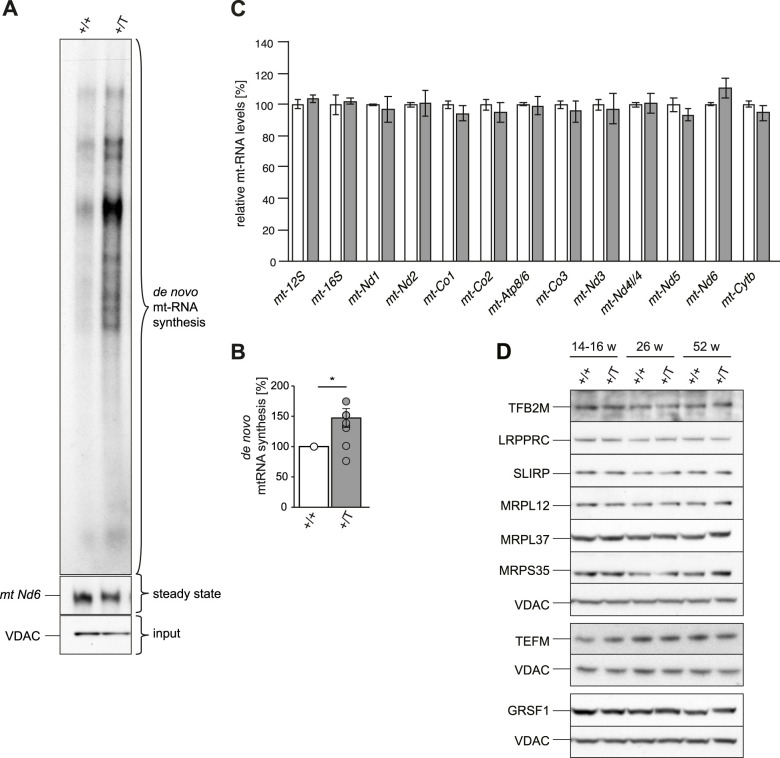
*Polrmt* overexpression increases transcription capacity. **(A)** Representative analysis of de novo-synthesized mitochondrial transcripts from the hearts of 14-wk-old WT (+/+) and *Polrmt*-overexpressing (+/T) mice. **(B)** Quantification of in organello synthesized mitochondrial transcripts. For each independent experiment, a +/+ and a littermate +/T were evaluated. Radioactive transcript signal intensity for +/+ and +/T was normalized to mitochondrial protein load (VDAC). The normalized +/+ signal was set to 100% and +/T is presented relative to +/+. n: 10 experiments. *P < 0.05, one-sample *t* test (μ = 100% corresponding to the +/+ normalization). **(C)** RNA-Seq of mt-rRNAs and mt-mRNAs on total RNA from isolated mitochondria from hearts of 14-wk-old +/+ and +/T mice. n: 3 per genotype. **(D)** Western blot of factors required for mitochondrial transcription or mt-RNA processing and in +/+ and +/T mice. Loading: VDAC; n: 3–4. GRSF1 was blotted in the same blot presented in Fig 3E, so VDAC is the same in both figures. Source data are available for this figure.

**Figure S6.**
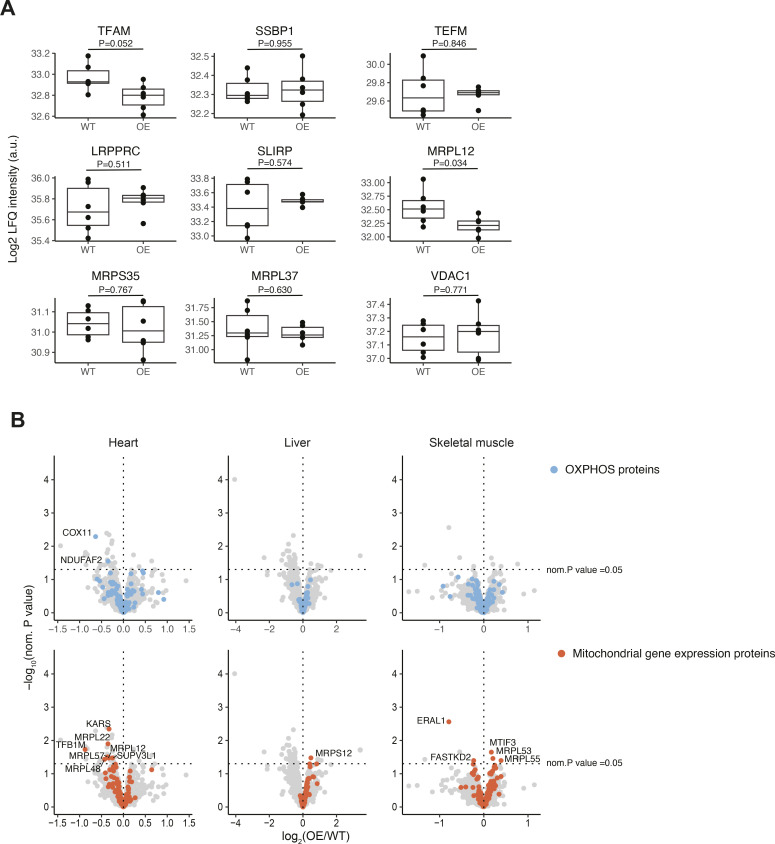
Label-free quantification (LFQ) mitochondrial proteome analysis from the heart, liver, and skeletal muscle. **(A)** Box plots of LFQ values for +/+ and +/T mice of some selected factors involved in mitochondrial gene expression. **(B)** Volcano plots of mitoproteomics analysis of 52-wk-old *Polrmt*-overexpressing (+/T) and WT (+/+) mice. n: 5/genotype. Proteins involved in OXPHOS and mitochondrial gene expression are highlighted.

Next, we studied the effect of *Polrmt* overexpression on mtDNA transcription in mouse hearts. First, we assessed de novo transcription in isolated heart mitochondria from *Polrmt*-overexpressing mice and found a significant increase of ∼50% (Fig 4A and B). There was no accumulation of specific transcription products, indicating that the processing of the polycistronic mt-RNAs is normal. This finding is consistent with reports that protein–protein interactions between TEFM and RNA-processing enzymes may link transcription elongation to RNA processing (Jiang et al, 2019). To assess the steady-state transcript levels of all mt-mRNAs and mt-rRNAs, we performed deep RNA sequencing (RNA-Seq) and found similar transcript levels in WT and *Polrmt*-overexpressing mice (Fig 4C) despite the strong increase in de novo transcription (Fig 4A and B). Interestingly, the *Polrmt*-overexpressing mice showed a trend toward elevated levels of the non-polyadenyated *mt-Nd6* transcript of the L-strand (Ruzzenente et al, 2012). Next, we investigated whether this discrepancy was accompanied by an imbalance in the protein levels of factors essential for posttranscriptional processes, which could explain why the increased transcription does not result in elevated steady-state levels of mt-mRNAs. We performed Western blots and label-free quantification proteomics on ultrapure mitochondria isolated from the heart, liver, and skeletal muscle. The volcano plots of the mitoproteomes of the heart, liver, and skeletal muscle show that only very few mitochondrial proteins were differentially expressed with a nominal *P*-value of 0.05; none of these differences were significant after multiple testing correction (Fig S6A and B). The steady-state protein levels of factors that are required for mtDNA transcription (TFAM, TEFM, TFB2M), mt-RNA processing and stability (G-rich sequence factor 1, GRSF1, LRPPRC, and SLIRP), and translation (mitochondrial ribosomal proteins MRPL37 and MRPS35) were not increased despite increased POLRMT levels (Figs 4D and S6). From the proteins involved in mtDNA gene expression or OXPHOS complex assembly, only the mitochondrial ribosomal protein, complex IV subunit COX11, and complex I assembly factor NDUFAF2 were down-regulated in the heart using nominal *P*-value. Thus, mouse hearts with increased POLRMT levels up-regulate mtDNA transcription (Fig 4A and B), whereas the steady-state mt-RNA levels and protein levels of other basal mtDNA gene expression machineries are not increased (Fig 4C and D).

### *Polrmt* overexpression increases *7S* RNA

**Figure 5.**
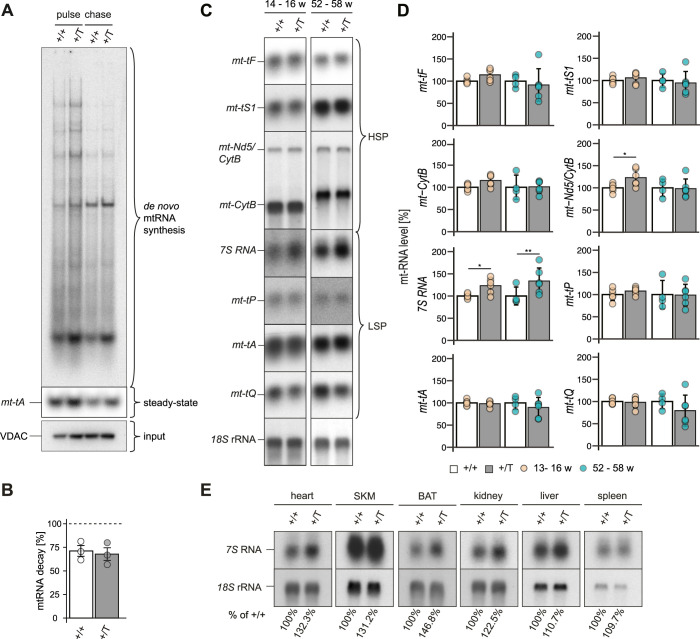
*Polrmt* overexpression increases LSP-promoter-proximal 7S RNA. **(A)** In organello synthesized mitochondrial transcripts from the hearts of 26-wk-old WT (+/+) and *Polrmt*-overexpressing (+/T) mice (pulse). The mRNA decay of newly synthesized transcripts was followed after 2 h (chase). Input: Western blot analysis VDAC on radiolabeled mitochondrial extracts. **(B)** Quantification of de novo synthesized mitochondrial transcripts normalized to VDAC and pulse signal; n: 3; grey:+/T, white:+/+. **(C)** Representative Northern blot analysis of mt-RNA levels in the heart of WT (+/+) and *Polrmt*-overexpressing (+/T) mice at different ages. **(D)** Quantification of mt-RNA levels from Northern blot analyses; normalization *18S* rRNA; *P < 0.05; ***P < 0.001; ANOVA; n: 4–6 per age. +/T and +/+ comparisons at different ages were tested within each age using a linear model with Tukey-adjusted pairwise tests. **(E)** Northern blot analysis of *7S* RNA levels in different tissues of a +/+ and +/T 52-wk-old mouse. n: 1 per genotype. Source data are available for this figure.

**Figure S7.**
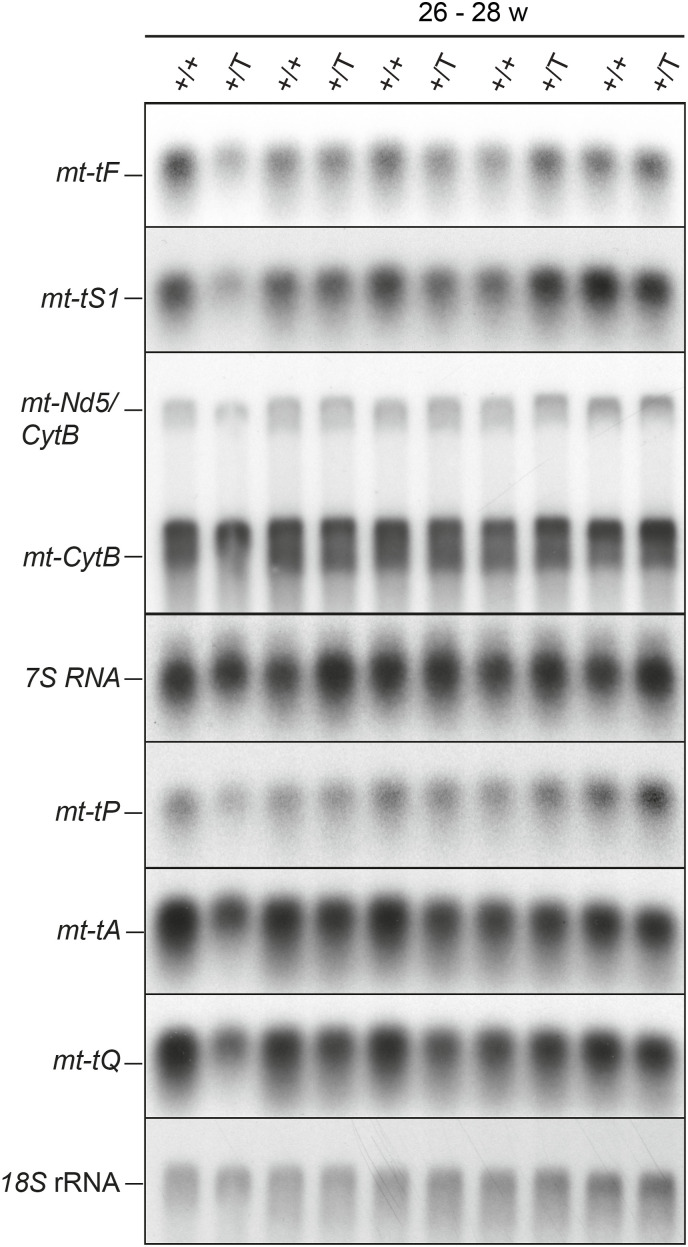
Northern blot at 26 w. Northern blot analysis of mt-RNA levels in the heart of WT (+/+) and *Polrmt*-overexpressing (+/T) mice at 26 wk.

We next tested whether the discrepancy between the normal steady-state transcript levels and the increased transcription in the *Polrmt*-overexpressing mice could be explained by decreased transcript stability. We followed the relative degradation rate of de novo labeled transcripts on in organello pulse-chase assays and found no evidence of increased RNA degradation in the *Polrmt*-overexpressing mitochondria (Fig 5A and B). We quantified the steady-state transcript levels of mt-tRNAs, the precursor transcript *mtNd5/CytB*, and the most promoter-proximal transcript generated from LSP, i.e., *7S* RNA (Fig 5C and D). Whereas most transcripts remained unchanged in the *Polrmt*-overexpressing mice, the most promoter-distal LSP transcript *mt-tQ* showed a mild decrease (Fig 5C and D). In contrast, we found a significant increase in *7S* RNA, the most promoter-proximal LSP transcript, across all the time points analyzed in the heart (Figs 5C and D and S7) and other mouse tissues (Fig 5E). Increased levels of *7S* RNA have previously been reported to correlate with increased transcription initiation (Cámara et al, 2011; Jiang et al, 2019), and our findings are therefore consistent with increased transcription initiation at LSP in the *Polrmt*-overexpressing mice.

### Co-overexpression of *Lrpprc* and *Polrmt* does not increase OXPHOS capacity

**Figure 6.**
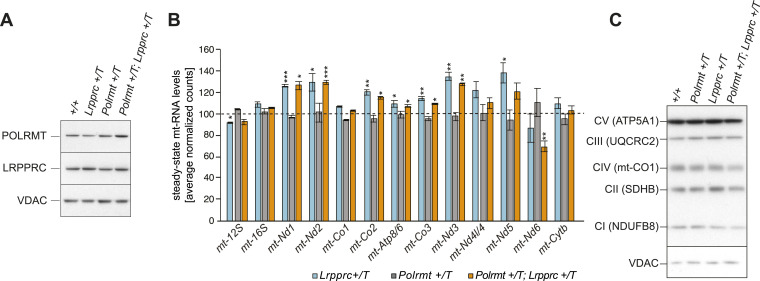
Stabilization of mitochondrial transcripts with *Lrpprc* overexpression does not result in a further increase of mt-RNAs in the *Polrmt*-overexpressing mice. **(A)** Western blot analysis of steady-state POLRMT and LRPPRC levels in mitochondrial extracts from the heart of WT (+/+), *Lrpprc*-overexpressing (*Lrpprc* +/T), *Polrmt*-overexpressing (*Polrmt* +/T), and *Polrmt* and *Lrpprc *double overexpressing (*Polrmt +/T*; *Lrpprc* +/T) mice. Loading: VDAC. **(B)** RNA-Seq of mt-rRNAs and mt-mRNAs on total RNA from hearts of 14-wk-old *Lrpprc* +/T, *Polrmt* +/T, and *Polrmt +/T*; *Lrpprc* +/T mice. Data are normalized to +/+, represented by the dotted line; n: 3 per genotype. **(C)** Representative Western blot of OXPHOS subunit levels in isolated mitochondria from heart at different ages; loading: VDAC. n: 3 per genotype. Source data are available for this figure.

Because protein levels of the other factors involved in mt-RNA metabolism, e.g., the mRNA-binding LRPPRC/SLIRP protein complex, were unchanged in the *Polrmt*-overexpressing mice (Fig 4D), we hypothesized that the excess of transcripts produced cannot be stabilized. To test this hypothesis, we generated mice simultaneously overexpressing *Lrpprc* (Harmel et al, 2013) and *Polrmt* (Fig 6A). *Lrpprc* overexpression increased the steady-state levels of mt-mRNAs without affecting OXPHOS capacity (Fig 6B and C), consistent with our previous results (Harmel et al, 2013). However, the combined ubiquitous overexpression of *Lrpprc* and *Polrmt* did not further increase the steady-state transcript levels in comparison with *Lrpprc* overexpression alone, and, consistently, OXPHOS protein levels remained normal (Fig 6C). Thus, our data support that the regulatory control of OXPHOS gene expression occurs downstream of transcription initiation and transcript stabilization.

## Discussion

In this study, we generated and characterized a mouse model overexpressing *Polrmt* to test whether increasing POLRMT under physiologic conditions modulates OXPHOS function and its effect on health. POLRMT is an essential mitochondrial protein required to initiate mtDNA replication and to transcribe the whole mitochondrial genome (Falkenberg et al, 2024). Isolated mitochondria from the *Polrmt*-overexpressing mice had higher levels of de novo transcription, showing that elevated POLRMT levels can increase mitochondrial transcription initiation. Because the protein levels of the other transcription factors were not changed in the POLRMT-overexpressing mice, the detection of elevated levels of transcription suggests that a ∼50% increase in POLRMT levels alone is sufficient to increase de novo transcription. We have previously reported that in vitro transcription is activated when increasing amounts of POLRMT are added and the levels of TFAM and TFB2M are held constant (Kühl et al, 2016). Furthermore, we previously determined that the molar ratios of POLRMT are much lower than TFAM and TFB2M in mouse liver (Harmel et al, 2013). Collectively, these findings argue that POLRMT is the limiting factor for transcription initiation. However, additional experiments with recombinant in vitro transcription systems and new mouse models with inducible *Polrmt* expression will be necessary to further study this issue. Our data conclusively show that a moderate ∼50% increase in POLRMT levels does not affect mitochondrial OXPHOS capacity, and is not pathogenic until 52 wk of age, the latest time point that we assessed. Only a handful of mitochondrial factors have been proposed to interact with POLRMT (Miranda et al, 2022), but none of them were found to be increased in different tissues of *Polrmt*-overexpressing mice, showing that their expression is not affected by moderately increased POLRMT levels. This finding raises the question of why POLRMT is up-regulated under several pathogenic conditions.

Transcription initiation from LSP generates (i) a short RNA replication primer, (ii) the *7S* RNA, and (iii) the full-length polycistronic transcript from the L-strand. The robust increase in *7S* RNA in the *Polrmt*-overexpressing mice argues that transcription initiation at LSP is increased. Although the protein levels of the components of the replisome are unchanged in the *Polrmt*-overexpressing mice, they may be present in excess and may therefore not be limiting for mtDNA replication (Jiang et al, 2021; Misic et al, 2022). It would be interesting to co-overexpress factors of the replisome in the *Polrmt*-overexpressing background because TWINKLE can be loaded at the 3′ end of *7S* DNA to promote full-length genomic replication and has been suggested to play a regulatory role in mtDNA replication (Milenkovic et al, 2013).

Despite the increase in de novo transcription and LSP-proximal transcripts, the steady-state levels of mature mitochondrial transcripts were surprisingly unchanged by *Polrmt* overexpression. Our data show that excess full-length transcripts are not produced in vivo in the *Polrmt* overexpressor mice. POLRMT produces two near-genomic length polycistronic transcripts that are processed and stabilized co-transcriptionally to generate the mature mt-RNAs. The stability of mt-rRNAs, mt-mRNAs, and mt-tRNAs, is mediated by different sets of nuclear-encoded proteins that are imported into the mitochondria; e.g., the LRPPRC–SLIRP complex promotes polyadenylation and stabilization of all mt-mRNAs, except *mt-Nd6* (Ruzzenente et al, 2012), whereas mitochondrial ribosomal proteins and assembly factors, which are generally synthesized in excess, stabilize mt-rRNAs (Park et al, 2007; Kühl et al, 2017; Bogenhagen et al, 2018). Increased transcription can therefore lead to different expression patterns of mtDNA encoded genes depending on post-transcriptional events, as exemplified by the *Lrpprc* knockout mice where mt-rRNAs and mt-tRNAs are increased, but mt-mRNAs are depleted (Ruzzenente et al, 2012). None of the mt-rRNAs, mt-tRNAs, or mt-mRNAs show significantly increased steady-state levels in the *Polrmt*-overexpressing mice, which shows that a moderate POLRMT increase on its own does not lead to a global increase in steady-state levels of mitochondrial transcripts. Furthermore, co-overexpressing *Lrpprc* and *Polrmt* did not increase mt-mRNAs more than *Lrpprc* overexpression alone arguing against the hypothesis that transcripts are generated in excess and not stabilized when *Polrmt* is overexpressed. Although our data do not exclude that protein levels of other factors acting at later steps of mitochondrial transcript stabilization are important, we did not find evidence of decreased transcript stability using pulse-chase in organello transcription assays in isolated mitochondria.

An alternative hypothesis to explain the discrepancy between increased transcription in isolated mitochondria and the unchanged steady-state transcript levels is that different transcription scenarios are occurring in vivo compared with ex vivo isolated mitochondria. A recent in vitro study in cultured human cells has shown that the *7S* RNA has a regulatory role in mitochondrial transcription by directly targeting and blocking the ability of POLRMT to initiate transcription (Zhu et al, 2022). The *7S* RNA interacts with POLRMT leading to POLRMT dimerization, which sequesters essential domains for promoter recognition and unwinding. Our data are consistent with this possible mechanism, and the high levels of *7S* RNA may be preventing the increased POLRMT molecules from engaging in full-length mtDNA transcription; thus, most transcripts remain unchanged under basal physiologic conditions. The increased transcription we report in the de novo transcription assays could be explained by a removal of the negative regulation of *7S* RNA on mitochondrial transcription in response to the ADP-stimulated respiration conditions in which this assay is performed. This raises the question of whether the increased capacity to up-regulate mitochondrial transcription can underlie the increased exercise capacity in mice when challenged. Further experiments will be required to assess the effect on OXPHOS in these mice after exercise.

Under non-pathologic conditions, we never obtained a founder mouse with more than one additional *Polrmt* gene inserted into the genome despite repeated efforts, suggesting that further increases in POLRMT levels might not be tolerated in vivo. Such dose-dependent toxicity has been shown to occur in vivo with TFAM, where moderate overexpression results in increased mtDNA copy number but unaltered mtDNA expression and health-status, whereas strong overexpression results in deficient OXPHOS and early postnatal lethality (Bonekamp et al, 2021). In human cell lines, overexpression of *POLRMT* has been reported to have inconsistent effects. In HeLa cells, strong overexpression of *POLRMT* with an N-terminal HA tag did not result in increased transcripts but resulted in decreased OXPHOS protein levels (Surovtseva & Shadel, 2013). However, in non-small lung cell cancer cells, overexpression of *POLRMT* from a lentiviral construct (10-fold mRNA and 2-3-fold POLRMT protein level increase) resulted in a 2–3-fold increase of mature mt-mRNAs (Zhou et al, 2021). The discrepant results in cell culture experiments may be attributed to differences in the used overexpression constructs and the obtained overexpression levels, in combination with different bioenergetic demands depending on the culture conditions. Interestingly, a strong overexpression of the mitochondrial RNA polymerase (*rpo41*) in fission yeast increased the mitochondrial transcription capacity and led to increased steady-state levels of mitochondrial transcripts and was shown to promote the survival of yeast cells from colonies that were exposed to cold (Kelly & Lehman, 1986; Masters et al, 1987; Jiang et al, 2011). The increased exercise capacity of the *Polrmt*-overexpressing mice suggests that the transgenic mice can adapt faster to higher energetic demands.

POLRMT is a key factor for mtDNA expression and replication and is therefore essential for OXPHOS biogenesis in mammalian cells. Using several mouse models, we characterized the molecular consequences of ubiquitous *Polrmt* overexpression in different tissues. We suggest that POLRMT is the limiting factor for mtDNA transcription in vivo, but that additional regulatory steps downstream of transcription initiation and transcript stability limit OXPHOS biogenesis. Increasing POLRMT levels did not result in any pathological phenotype but led to increased exercise capacity under stress conditions. Further experiments would be required to assess the effect on OXPHOS in these mice after exercise. Our data support a model where POLRMT increases transcription initiation, allowing rapid adaptation to changed energetic demands, e.g., in response to increased exercise, or in pathogenic states such as cancer.

## Materials and Methods

### Generation and genotyping of transgenic *Polrmt*-overexpressing mice

BAC clones containing a fragment of chromosome 10, including the entire *Polrmt* gene, were purchased from the C57Bl/6N BAC library of DNA Bank, RIKEN BioResource Center. The BAC clone BgN01-092D16 was modified by Red/ET recombination using the counter-selection BAC modification kit (Genebridges). A silent point mutation 420G>T was introduced into exon 3 leading to a unique HindIII restriction site. Positive clones were verified by PCR followed by HindIII restriction digest, Sanger sequencing, and Southern blotting. The modified BAC was purified via a cesium chloride gradient and injected into the pronucleus of fertilized oocytes as described in Milenkovic et al (2013). Founders (+/BAC-*Polrmt*) were identified by PCR and restriction enzyme analysis of genomic DNA to detect gain of the HindlII site in the *Polrmt* gene. Tail DNA from offspring was genotyped for the presence of the BAC transgene by analyzing 100 ng of tail DNA with the GoTaq PCR reaction kit (Promega) according to the manufacturer’s instruction by adding forward primer 5′-GAG​GCT​CGG​GTG​CGG​CAG​CTC -3′ and reverse primer 5′-GTG​CAG​TGT​GAG​CAC​CTG​CTG​TC-3′ for PCR with an initial denaturation for 3 min at 95°C, followed by 40 cycles for 30 s at 95°C, 30 s at 60°C, and 45 s at 72°C. The reaction was ended with extension for 10 min at 72°C. Mice were maintained heterozygous on an inbred C57BL/6N background.

### Ethical statement

Animals were housed in individually ventilated cages under specific pathogen–free conditions with constant temperature (21°C) and humidity (50–60%) and a 12-h light/dark cycle. All mice were fed commercial rodent chow and provided with acidified water ad libitum. Mice were euthanized by cervical dislocation. The health status of the animals is specific pathogen free according to the Federation of the European Laboratory Animal Science Association (FELASA) recommendations. All animal procedures were conducted in accordance with European, national and institutional guidelines and protocols (no.: AZ.: 84-02.05.50.15.004, AZ.: 84-02.04.2015.A103 and 84-02.04.2016.A420) were approved by the Landesamt für Natur, Umwelt und Verbraucherschutz, Nordrhein-Westfalen, Germany.

### Exercise challenges and phenotyping protocol

#### Treadmill

Mice were placed on a treadmill (TSE Systems) for a 5-min habituation. After a 10-min warm-up phase at 0.1 m/sec, the speed was increased continuously by 0.02 m/min. If the mice did not keep up with the treadmill speed, they were exposed to a mild electric shock (0.3 mA). The distance was recorded until mice received three consecutive shocks.

#### Indirect calorimetry

Indirect calorimetry and home cage locomotor activity were monitored for singly housed mice in purpose-built cages (Phenomaster, TSE Systems). Parameters were measured for 48 h after at least 4 d of acclimatization. For the calculation of the respiratory exchange ratio, the volumes of consumed O_2_ and produced CO_2_ were normalized to lean body weight.

#### Running wheel

Voluntary running was monitored for 2 wk in individually housed mice using wireless mouse running wheels (Med Associates Inc.).

#### Body composition

Body fat and lean mass content were measured in vivo by nuclear magnetic resonance using the minispec LF50H (Bruker Optics).

### DNA isolation, Southern blot analysis, and mtDNA quantification

For genotyping and pyrosequencing, DNA from tail or ear-clip biopsies DNA was extracted using chloroform and ethanol precipitation. For Southern blotting or qPCR, total DNA was isolated from mouse tissues using the DNeasy Blood & Tissue Kit (QIAGEN) as previously described (Kühl et al, 2016). Briefly, snap frozen tissues were ground in a cold mortar. About 20 mg of ground tissue was used to extract DNA using the blood and tissue kit or the Gentra Puregene tissue kit (QIAGEN) following the manufacturer’s instructions. All samples used for Southern blotting and qPCR were treated with RNase (QIAGEN). For Southern blot analysis, total DNA (3–10 μg) was digested with SacI endonuclease, fragments were separated by agarose gel electrophoresis, transferred to nitrocellulose membranes (Hybond-N^+^ membranes, GE Healthcare), and hybridized with alpha-^32^P-dCTP–labeled probes to detect total mtDNA (pAM1) using nuclear DNA (*18S* rDNA) as loading control. mtDNA was also measured by semiquantitative PCR carried out on 4 ng of total DNA in a 7900HT Real-Time PCR System (Applied Biosystems) using TaqMan probes specific for the mt-*Co1*, *mt-Co2*, mt-*Cytb*, mt-*Nd5*, mt-*Nd6*, and *18S* genes (Applied Biosystems).

### Pyrosequencing

Quantification of *Polrmt* gene dosage was performed on tail DNA using a PyroMark-Q24 pyrosequencer (QIAGEN). Allele quantification assay was developed using PyroMark assay design software v. 2.0 (QIAGEN). A single PCR reaction was used to amplify a 192-bp fragment spanning the c.420G>T mutation site, using a biotinylated primer ([Btn]TCTTGCTTGGCTGCAGGTAG) and a non-biotinylated primer (AGA​GGC​GCC​AAA​AGG​AAG​TT). PCR products were combined with distilled water, PyroMark binding buffer (QIAGEN), and 1 μl Streptavidin Sepharose high performance beads (GE Healthcare). Next, the PCR products were purified and denatured using a PyroMark Q24 vacuum workstation (QIAGEN). Sequencing was performed with PyroMark Gold Q24 reagents according the manufacturer’s instructions using the sequencing primer (CAA​GAT​CTG​GAA​CAA​GAA). Relative allele frequencies were calculated using the PyroMark-Q24 Advance v.3.0.0 software (QIAGEN).

### RT–PCR, qRT-PCR, and Northern blot analysis

RNA was isolated by using the miRNeasy Mini Kit (QIAGEN). Reverse transcription PCR (RT–PCR) was carried out after cDNA synthesis using the High-Capacity cDNA Reverse Transcription Kit (Applied Biosystems). Real-time quantitative reverse transcription PCR (qRT-PCR) was performed using the Taqman 2x Universal PCR master mix, No Amperase UNG (Applied Biosystems). The quantity of transcripts was normalized to the TATA-binding protein (*Tbp*) RNA as a reference gene transcript. For Northern blotting, 1–2 μg of total RNA was denatured in RNA sample loading buffer (Sigma-Aldrich), separated on 1.2 or 1.8% formaldehyde-MOPS agarose gels before transferring onto Hybond-N^+^ membranes (GE Healthcare) overnight. After UV crosslinking, the blots were hybridized with various probes at 42°C or 65°C in RapidHyb buffer (Amersham) and thereafter washed in 2x and 0.2x SSC/0.1% SDS before exposure to film. Mitochondrial probes used for visualization of mt-mRNA and mt-rRNA levels were restriction fragments labeled with α-^32^P-dCTP and a random priming kit (Agilent). Different mitochondrial tRNAs and *7S* RNA were detected using specific oligonucleotides labeled with γ-^32^P-ATP. Radioactive signals were detected by autoradiography.

### De novo transcription and replication assays

In organello transcription and replication assays were performed on mitochondria isolated from mouse hearts by differential centrifugation as described before (Agaronyan et al, 2015; Kühl et al, 2016; Jiang et al, 2021; Misic et al, 2022). In organello transcription assays were carried out as previously reported (Lagouge et al, 2015). For each in organello replication assay, 1 mg of purified mitochondria were washed in 1 ml of incubation buffer (10 mM Tris, pH 7.4, 25 mM sucrose, 75 mM sorbitol, 100 mM KCl, 10 mM K_2_HPO_4_, 50 μM EDTA, 5 mM MgCl_2_, 10 mM glutamate, 2.5 mM malate, 1 mg/ml BSA, and 1 mM ADP) and resuspended in 500 μl incubation buffer supplemented with 50 μM of dCTP, dTTP, dGTP, and 20 μCi of α-^32^P-dATP (PerkinElmer) and incubated for 2 h at 37°C as reported (Matic et al, 2018). For chase experiments, radiolabeled mitochondria were washed and incubated for 2 h in incubation buffer without radioactivity. After incubation, mitochondria were washed three times in 10 mM Tris, pH 6.8, 0.15 mM MgCl_2_, and 10% glycerol. An aliquot of radiolabelled mitochondria was collected for immunoblotting with the VDAC (Millipore) or SDHA (Invitrogen) as a loading control. MtDNA was isolated by phenol/chloroform extractions or by Puregene Core Kit A (QIAGEN) and radiolabeled replicated DNA was analyzed by Southern blotting and visualized by autoradiography. Quantifications of transcript levels were performed using the program Multi Gauge with images generated from a PhospoImager instrument.

### RNA sequencing

RNA was isolated from crude heart mitochondria using the miRNeasy Mini Kit (QIAGEN), and the concentration, purity, and integrity were confirmed using a BioAnalyser. RNA sequencing libraries were constructed using the Illumina TruSeq Sample Prep Kit. Paired end deep sequencing of the mitochondrial RNAs was performed on an Illumina MiSeq according to the manufacturer’s instructions. RNA-Seq was performed on mitochondrial RNA from three biological replicates of each genotype: wild-type, *Polrmt*-overexpressing, *Lrpprc*-overexpressing, and *Polrmt* and *Lrpprc* double overexpressing mice aged 14 wk. The alignment to the *Mus musculus* reference genome (GRCm38) was performed using HISAT2 version 2.0.5 (--dta) (Kim et al, 2019). Alignment files were sorted and indexed with SAMtools version 1.3.1 (Li et al, 2009). Transcript abundances were estimated with StringTie version 1.2.4 (−e-B) (Pertea et al, 2016) and raw reads count matrices at gene level were extracted with the included prepDE.py script on Python version 2.7.6. Differential gene expression was performed with DESeq version 1.14.1 (Love et al, 2014) after filtering of low abundance genes. Genes with an adjusted *P*-value < 0.05 were considered statistically significant. 

### Western blots and antisera

For crude mitochondria isolation, mouse tissues were homogenized using a Potter S homogenizer (Sartorius) in mitochondrial isolation buffer (320 mM sucrose, 1 mM EDTA, and 10 mM Tris–HCl, pH 7.4) containing complete protease inhibitor cocktail (Roche) followed by two rounds of differential centrifugation. Isolation of crude mitochondria from skeletal muscle was performed as previously described (Frezza et al, 2007). The protein concentration of protein samples was determined using Bradford reagent (Bio-Rad) and BSA as a standard. Proteins were separated by SDS–PAGE (using 4–12% Tris-glycine gels, Invitrogen) and then transferred onto polyvinylidene difluoride membranes (GE Healthcare) using wet tank blotting (25 mM Tris, 192 mM glycine, and 20% ethanol) at 4°C at 400 mA for 2 h or at 80 mA overnight. Immunoblotting was performed according to standard techniques using enhanced chemiluminescence (Immun-Star HRP Luminol/Enhancer from Bio-Rad). The following antibodies were used: Total OXPHOS Rodent WB Antibody Cocktail containing NDUFB8 (Complex I), SDHB (Complex II), mt-COI (CIV), UQRC2 (CIII), and ATP5A1 (CV) (ab110413; Abcam), SDHA (Invitrogen), TFAM (Abcam), VDAC (porin) from Mitoscience, and GRSF1, SSBP1, MRPL12, MRPS35, and MRPL37 from Sigma-Aldrich. Further, polyclonal antisera were used to detect TFB2M, SLIRP, LRPPRC, TWINKLE, TEFM, and POLRMT proteins (Ruzzenente et al, 2012; Milenkovic et al, 2013; Kühl et al, 2014; Lagouge et al, 2015; Jiang et al, 2019). Western blots were quantified with Fiji Image J, and each blot was normalized to the average or WT run on the same blot.

### Ultrapure mitochondria isolation, peptide digestion, and cleanup for label-free mass spectrometry

Mitochondria were isolated from mouse hearts using differential centrifugation as previously reported (Kühl et al, 2016). For proteomic analysis, crude mitochondrial pellets were washed in 1xM buffer (220 mM mannitol, 70 mM sucrose, 5 mM Hepes, pH 7.4, 1 mM EGTA, pH 7.4; pH was adjusted with potassium hydroxide; supplemented with EDTA-free complete protease inhibitor cocktail and PhosSTOP Tablets [Roche]) and purified on a Percol density gradient (12%:19%:40% prepared with buffer 2xM) via centrifugation in a SW41 Ti Swinging-Bucket rotor (Beckman) at 15,500 rpm at 4°C for 1 h in a Beckman Coulter Optima L-100 XP ultracentrifuge using 14 × 89 mm Ultra-Clear centrifuge tubes (Beckman Instruments Inc.) as previously described (Kühl et al, 2017). Mitochondria were harvested at the interphase between 19% and 40%, washed three times with buffer 1xM, and the mitochondrial pellets were frozen at −80°C. Purified frozen mitochondria pellets were suspended in lysis buffer (6 M guanidinium chloride, 10 mM Tris(2-carboxyethyl)phosphine hydrochloride, 40 mM chloroacetamide, and 100 mM Tris–HCl) (Kulak et al, 2014). After complete lysis, the samples were diluted 1:10 in 20 mM Tris–HCL, pH 8.0, and 80 μg of protein was mixed with 3 μg of trypsin gold (Promega) and incubated overnight at 37°C to achieve complete digestion. The peptides were cleaned with home-made STAGEtips (Rappsilber et al, 2003) (Empore Octadecyl C18; 3 M) and eluted in 60% acetonitrile/0.1% formic acid buffer. The samples were dried in a SpeedVac apparatus (Eppendorf concentrator plus 5305) at 45°C, and the peptides were suspended with 0.1% formic acid. Approximately 1.5 μg of peptides was analyzed by LC–MS/MS.

### LC–MS/MS analysis

For mass spectrometric analysis, the peptides were separated on a 25-cm, 75-μm internal diameter PicoFrit analytical column (New Objective, Part No. PF7508250) packed with 1.9 μm ReproSil-Pur 120 C18-AQ medium (Mat. No. r119.aq; Dr. Maisch) using an EASY-nLC 1000 or EASY-nLC 1200 (Thermo Fisher Scientific). The column was maintained at 50°C. Buffer A and B were 0.1% formic acid in water and 0.1% formic acid in acetonitrile, respectively. For the analysis using the EASY-nLC 1,200 system, buffer B was 80% acetonitrile, 0.1% formic acid. Peptides were separated on a segmented gradient from 2% to 5% buffer B for 10 min, from 5% to 20% buffer B for 100 min, from 20% to 25% buffer B for 10 min, and from 25% to 45% buffer B for 10 min at 200 nl/min (EASY-nLC 1,000). Using the EASY-nLC 1200 system, peptides were separated on a segmented gradient from 3% to 6% buffer B for 10 min, from 6% to 25% buffer B for 100 min, from 25% to 31% buffer B for 10 min, and from 31% to 60% buffer B for 10 min, at 200 nl/min. Eluting peptides were analyzed on a QExactive HF mass spectrometer (Thermo Fisher Scientific). Peptide precursor mass to charge ratio (m/z) measurements (MS1) were carried out at 60,000 resolution in the 300–1,800 m/z range. The top 10 most intense precursors with charge state from two to seven only were selected for HCD fragmentation using 25% collision energy. The m/z of the peptide fragments (MS2) were measured at 30,000 resolution using an AGC target of 2 × 10^5^ and 80 ms maximum injection time. Upon fragmentation, the precursors were put on an exclusion list for 45 sec. Peptides from the three different tissues were analyzed in a single run.

### LC–MS/MS data analysis

The raw data were analyzed with MaxQuant version 1.4.1.2 using the integrated Andromeda search engine (Cox et al, 2011). Peptide fragmentation spectra were searched against the canonical and isoform sequences of the mouse reference proteome (proteome ID UP000000589, August 2015 from UniProt). The database was complemented with 245 sequences of contaminating proteins by MaxQuant. For the analysis methionine oxidation and protein N-terminal acetylation were set as variable modifications. The digestion parameters were set to “specific” and “Trypsin/P,” allowing for cleavage after lysine and arginine also when followed by proline. The minimum number of peptides and razor peptides for protein identification was 1; the minimum number of unique peptides was 0. Protein identification was performed at a peptide spectrum matches and protein false discovery rate (FDR) of 0.01. The “second peptide” option was on to identify co-fragmented peptides. Successful identifications were transferred between the different raw files using the “Match between runs” option, using a match time window of 0.7 min. Label-free quantification (LFQ) (Cox et al, 2014) was performed using an LFQ minimum ratio count of 2.

### Protein quantification analysis

Analysis of the label-free quantification (LFQ) results was carried out using the Perseus computation platform (Tyanova et al, 2016), version 1.5.0.0 and R, version 3.3.0 (R Development Core Team, 2010). For the analysis, LFQ intensity values were loaded in Perseus and all identified proteins marked as “Reverse,” “Only identified by site,” and “Potential contaminant” were removed. The corresponding +/+ and the +/T genotypes were loaded separately in Perseus, the LFQ intensity values were log_2_ transformed and all proteins that contained less than two to five missing values in one of the groups (+/+ or +/T) were removed. Missing values in the resulting subset of proteins were imputed with a width of 0.3 and down shift of 1.8. Imputed LFQ intensities were loaded into R where a two-sided moderated *t* test analysis was performed using limma, version 3.30.13 (Cox et al, 2011). Proteins with an adjusted *P*-value (“BH” correction) of less than 0.05 were designated as differentially expressed. Our list of differentially expressed proteins was combined with the pathway annotations from MitoCarta3.0 (Rath et al, 2021).

### Bioenergetic determinations

Mitochondrial oxygen consumption flux was measured at 37°C using 65–125 μg of crude mitochondria diluted in 2.1 ml of mitochondrial respiration buffer (120 mM sucrose, 50 mM KCl, 20 mM Tris–HCl, 4 mM KH_2_PO_4_, 2 mM MgCl_2_, and 1 mM EGTA, pH 7.2) in an Oxygraph-2k (Oroboros Instruments, Innsbruck, Austria). The oxygen consumption rate was measured using either 10 mM pyruvate, 10 mM glutamate, and 5 mM malate or 10 mM succinate and 10 nM rotenone. Oxygen consumption was assessed in the phosphorylating state with 1 mM ADP (state 3) or non-phosphorylating state by adding 2.5 μg/ml oligomycin (state 4). Respiration was uncoupled by successive addition of carbonyl cyanide m-chlorophenyl hydrazone (CCCP) up to 3 μM to reach maximal respiration.

To measure mitochondrial respiratory chain complex activities 15–50 μg of mitochondria were diluted in phosphate buffer (KH_2_PO_4_ 50 mM, pH 7.4), followed by spectrophotometric analysis of isolated respiratory chain complex activities at 37°C, using a Hitachi UV-3600 spectrophotometer. To follow citrate synthase activity, increase in absorbance at 412 nm was recorded after addition of acetyl-CoA (0.1 mM), oxaloacetate (0.5 mM) and DTNB (0.1 mM). Succinate dehydrogenase (SDH) activity was measured at 600 nm after the addition of 10 mM succinate, 35 μM dichlorphenolindophenol (DCPIP) and 1 mM KCN. NADH dehydrogenase activity was determined at 340 nm after the addition of 0.25 mM NADH, 0.25 mM decylubiquinone, and 1 mM KCN and controlling for rotenone sensitivity. Cytochrome *c* oxidase activity was measured by standard TMPD ascorbate/KCN sensitive assays. To assess the ATPase activity, 65 μg/ml frozen isolated mitochondria was incubated at 37°C in triethanolamine 75 mM, MgCl_2_ 2 mM, pH 8.9. Mitochondria were preincubated 2 min with alamethicin 10 μg/ml before addition of 2 mM of ATP. The samples were removed every 2 min and precipitated in 7% HClO_4_, 25 mM EDTA (50 μl). Phosphate was quantified by incubating an aliquot in 1 ml molybdate 5.34 mM, ferrous sulfate (28.8 mM), and H_2_SO_4_ 0.75 N for 2 min. The absorbance was assessed at 600 nm. In parallel, oligomycin (2.5 μg per ml protein) was added to the mitochondrial suspension to determine the oligomycin insensitive ATPase activity. Each activity was normalized to mg protein by using the Lowry-based Bio-Rad protein DC kit. All chemicals were obtained from Sigma-Aldrich.

### Statistical analysis

Each mouse was considered an independent biological replicate (n), and repeated measurements from the same animal were treated as technical replicates. Unless otherwise indicated, ≥3 biological replicates from the transgenic mouse strain and their respective age-matched control mice were used for all experiments. Statistical analyses for RNA-Seq were performed as described above. Linear modeling was used to assess the effects of Genotype, Age, and their interaction (lm(output.variable ∼ Genotype * Age)) on *Polrmt* transcript levels, tissue respirometry, mitochondrial transcripts measured by Northern blot, and mtDNA levels measured by Southern blot. Estimated marginal means (EMMs) were computed using the emmeans package in R to evaluate group differences. Pairwise comparisons between genotypes were performed within each age-group using Tukey’s method to adjust for multiple comparisons. Statistical testing for POLRMT protein levels was performed using a two-sided *t* test. In organello experiments were analyzed using a one-sample, two-tailed *t* test with μ:100 as each experiment was normalized as percentage of the WT control that was run simultaneously. Statistical analyses were performed in Excel or R Studio version 1.1.383. Data visualization in R was performed using ggplot2 version 2.2.1. The definition of center and precision measures, and *P*-values are provided in the figure legends. *P* < 0.05 was considered significant.

## Supplementary Material

Reviewer comments

## Data Availability

Proteomic datasets are available at ProteomeXchange with identifier PXD068527. RNA-seq are available at GEO (accession number: GSE307758). Any additional information required to reanalyze the data reported in this article is available from the lead contact upon request.

## References

[bib1] Agaronyan K, Morozov YI, Anikin M, Temiakov D (2015) Mitochondrial biology. Replication-transcription switch in human mitochondria. Science 347: 548–551. 10.1126/science.aaa0986 25635099 PMC4677687

[bib2] Bogenhagen DF, Ostermeyer-Fay AG, Haley JD, Garcia-Diaz M (2018) Kinetics and mechanism of mammalian mitochondrial ribosome assembly. Cell Rep 22: 1935–1944. 10.1016/j.celrep.2018.01.066 29444443 PMC5855118

[bib3] Bonekamp NA, Peter B, Hillen HS, Felser A, Bergbrede T, Choidas A, Horn M, Unger A, Di Lucrezia R, Atanassov I, (2020) Small-molecule inhibitors of human mitochondrial DNA transcription. Nature 588: 712–716. 10.1038/s41586-020-03048-z 33328633

[bib4] Bonekamp NA, Jiang M, Motori E, Garcia Villegas R, Koolmeister C, Atanassov I, Mesaros A, Park CB, Larsson N-G (2021) High levels of TFAM repress mammalian mitochondrial DNA transcription in vivo. Life Sci Alliance 4: e202101034. 10.26508/lsa.202101034 34462320 PMC8408345

[bib5] Bralha FN, Liyanage SU, Hurren R, Wang X, Son MH, Fung TA, Chingcuanco FB, Tung AYW, Andreazza AC, Psarianos P, (2015) Targeting mitochondrial RNA polymerase in acute myeloid leukemia. Oncotarget 6: 37216–37228. 10.18632/oncotarget.6129 26484416 PMC4741925

[bib6] Cámara Y, Asin-Cayuela J, Park CB, Metodiev MD, Shi Y, Ruzzenente B, Kukat C, Habermann B, Wibom R, Hultenby K, (2011) MTERF4 regulates translation by targeting the methyltransferase NSUN4 to the mammalian mitochondrial ribosome. Cell Metab 13: 527–539. 10.1016/j.cmet.2011.04.002 21531335

[bib7] Chaudhary S, Ganguly S, Palanichamy JK, Singh A, Bakhshi R, Jain A, Chopra A, Bakhshi S (2021) PGC1A driven enhanced mitochondrial DNA copy number predicts outcome in pediatric acute myeloid leukemia. Mitochondrion 58: 246–254. 10.1016/j.mito.2021.03.013 33812061

[bib8] Cox J, Michalski A, Mann M (2011) Software lock mass by two-dimensional minimization of peptide mass errors. J Am Soc Mass Spectrom 22: 1373–1380. 10.1007/s13361-011-0142-8 21953191 PMC3231580

[bib9] Cox J, Hein MY, Luber CA, Paron I, Nagaraj N, Mann M (2014) Accurate proteome-wide label-free quantification by delayed normalization and maximal peptide ratio extraction, termed MaxLFQ. Mol Cell Proteomics 13: 2513–2526. 10.1074/mcp.m113.031591 24942700 PMC4159666

[bib10] Falkenberg M (2018) Mitochondrial DNA replication in mammalian cells: Overview of the pathway. Essays Biochem 62: 287–296. 10.1042/EBC20170100 29880722 PMC6056714

[bib11] Falkenberg M, Larsson N-G, Gustafsson CM (2024) Replication and transcription of human mitochondrial DNA. Annu Rev Biochem 93: 47–77. 10.1146/annurev-biochem-052621-092014 38594940

[bib12] Frezza C, Cipolat S, Scorrano L (2007) Organelle isolation: Functional mitochondria from mouse liver, muscle and cultured fibroblasts. Nat Protoc 2: 287–295. 10.1038/nprot.2006.478 17406588

[bib13] García-Villegas R, Odenthal F, Giannoula Y, Bonekamp NA, Kühl I, Park CB, Spåhr H, Motori E, Levander F, Larsson N-G (2025) In vivo composition of the mitochondrial nucleoid in mice. Biochim Biophys Acta Mol Cell Res 1872: 119955. 10.1016/j.bbamcr.2025.119955 40246179

[bib14] Gohil VM, Nilsson R, Belcher-Timme CA, Luo B, Root DE, Mootha VK (2010) Mitochondrial and nuclear genomic responses to loss of LRPPRC expression. J Biol Chem 285: 13742–13747. 10.1074/jbc.M109.098400 20220140 PMC2859537

[bib15] Goldstein RE (1990) Exercise capacity. In Clinical Methods: The History, Physical, and Laboratory Examinations, Walker HK, Hall WD, Hurst JW (eds). Boston, MA: Butterworths. 21250045

[bib16] Harmel J, Ruzzenente B, Terzioglu M, Spåhr H, Falkenberg M, Larsson N-G (2013) The leucine-rich pentatricopeptide repeat-containing protein (LRPPRC) does not activate transcription in mammalian mitochondria. J Biol Chem 288: 15510–15519. 10.1074/jbc.M113.471649 23599432 PMC3668712

[bib75] Herbine K, Nayak AR, Zamudio-Ochoa A, Temiakov D (2025) Structural basis for promoter recognition and transcription factor binding and release in human mitochondria. Mol Cell 85. 10.1016/j.molcel.2025.06.016 PMC12313153 40712587

[bib17] Hillen HS, Parshin AV, Agaronyan K, Morozov YI, Graber JJ, Chernev A, Schwinghammer K, Urlaub H, Anikin M, Cramer P, (2017) Mechanism of transcription anti-termination in human mitochondria. Cell 171: 1082–1093.e13. 10.1016/j.cell.2017.09.035 29033127 PMC5798601

[bib18] Hillen HS, Temiakov D, Cramer P (2018) Structural basis of mitochondrial transcription. Nat Struct Mol Biol 25: 754–765. 10.1038/s41594-018-0122-9 30190598 PMC6583890

[bib19] Holzmann J, Frank P, Löffler E, Bennett KL, Gerner C, Rossmanith W (2008) RNase P without RNA: Identification and functional reconstitution of the human mitochondrial tRNA processing enzyme. Cell 135: 462–474. 10.1016/j.cell.2008.09.013 18984158

[bib20] Isaac RS, Tullius TW, Hansen KG, Dubocanin D, Couvillion M, Stergachis AB, Churchman LS (2024) Single-nucleoid architecture reveals heterogeneous packaging of mitochondrial DNA. Nat Struct Mol Biol 31: 568–577. 10.1038/s41594-024-01225-6 38347148 PMC11370055

[bib21] Jiang H, Sun W, Wang Z, Zhang J, Chen D, Murchie AIH (2011) Identification and characterization of the mitochondrial RNA polymerase and transcription factor in the fission yeast Schizosaccharomyces pombe. Nucleic Acids Res 39: 5119–5130. 10.1093/nar/gkr103 21357609 PMC3130274

[bib22] Jiang S, Koolmeister C, Misic J, Siira S, Kühl I, Silva Ramos E, Miranda M, Jiang M, Posse V, Lytovchenko O, (2019) TEFM regulates both transcription elongation and RNA processing in mitochondria. EMBO Rep 20: e48101. 10.15252/embr.201948101 31036713 PMC6549021

[bib23] Jiang M, Xie X, Zhu X, Jiang S, Milenkovic D, Misic J, Shi Y, Tandukar N, Li X, Atanassov I, (2021) The mitochondrial single-stranded DNA binding protein is essential for initiation of mtDNA replication. Sci Adv 7: eabf8631. 10.1126/sciadv.abf8631 34215584 PMC11057760

[bib24] Kelly J, Lehman I (1986) Yeast mitochondrial RNA polymerase. Purification and properties of the catalytic subunit. J Biol Chem 261: 10340–10347. 10.1016/s0021-9258(18)67529-5 3525543

[bib25] Kim D, Paggi JM, Park C, Bennett C, Salzberg SL (2019) Graph-based genome alignment and genotyping with HISAT2 and HISAT-genotype. Nat Biotechnol 37: 907–915. 10.1038/s41587-019-0201-4 31375807 PMC7605509

[bib26] Kühl I, Kukat C, Ruzzenente B, Milenkovic D, Mourier A, Miranda M, Koolmeister C, Falkenberg M, Larsson N-G (2014) POLRMT does not transcribe nuclear genes. Nature 514: E7–E11. 10.1038/nature13690 25297440

[bib27] Kühl I, Miranda M, Posse V, Milenkovic D, Mourier A, Siira SJ, Bonekamp NA, Neumann U, Filipovska A, Polosa PL, (2016) POLRMT regulates the switch between replication primer formation and gene expression of mammalian mtDNA. Sci Adv 2: e1600963. 10.1126/sciadv.1600963 27532055 PMC4975551

[bib28] Kühl I, Miranda M, Atanassov I, Kuznetsova I, Hinze Y, Mourier A, Filipovska A, Larsson N-G (2017) Transcriptomic and proteomic landscape of mitochondrial dysfunction reveals secondary coenzyme Q deficiency in mammals. Elife 6: e30952. 10.7554/eLife.30952 29132502 PMC5703644

[bib29] Kukat C, Davies KM, Wurm CA, Spåhr H, Bonekamp NA, Kühl I, Joos F, Polosa PL, Park CB, Posse V, (2015) Cross-strand binding of TFAM to a single mtDNA molecule forms the mitochondrial nucleoid. Proc Natl Acad Sci U S A 112: 11288–11293. 10.1073/pnas.1512131112 26305956 PMC4568684

[bib78] Kulak NA, Pichler G, Paron I, Nagaraj N, Mann M (2014) Minimal, encapsulated proteomic-sample processing applied to copy number estimation in eukaryotic cells. Nat Methods 11: 319–324. 10.1038/nmeth.2834 24487582

[bib30] Lagouge M, Mourier A, Lee HJ, Spåhr H, Wai T, Kukat C, Silva Ramos E, Motori E, Busch JD, Siira S, (2015) SLIRP regulates the rate of mitochondrial protein synthesis and protects LRPPRC from degradation. PLoS Genet 11: e1005423. 10.1371/journal.pgen.1005423 26247782 PMC4527767

[bib31] Larsson N-G (2010) Somatic mitochondrial DNA mutations in mammalian aging. Annu Rev Biochem 79: 683–706. 10.1146/annurev-biochem-060408-093701 20350166

[bib32] Larsson N-G, Wang J, Wilhelmsson H, Oldfors A, Rustin P, Lewandoski M, Barsh GS, Clayton DA (1998) Mitochondrial transcription factor A is necessary for mtDNA maintenance and embryogenesis in mice. Nat Genet 18: 231–236. 10.1038/ng0398-231 9500544

[bib68] Li H, Handsaker B, Wysoker A, Fennell T, Ruan J, Homer N, Marth G, Abecasis G, Durbin R (2009) The sequence alignment/map format and SAMtools. Bioinformatics 25: 2078–2079. 10.1093/bioinformatics/btp352 19505943 PMC2723002

[bib33] Li X, Yao L, Wang T, Gu X, Wu Y, Jiang T (2023) Identification of the mitochondrial protein POLRMT as a potential therapeutic target of prostate cancer. Cell Death Dis 14: 665. 10.1038/s41419-023-06203-2 37816734 PMC10564732

[bib70] Love MI, Huber W, Anders S (2014) Moderated estimation of fold change and dispersion for RNA-seq data with DESeq2. Genome Biol 15: 550. 10.1186/s13059-014-0550-8 25516281 PMC4302049

[bib35] Masters BS, Stohl LL, Clayton DA (1987) Yeast mitochondrial RNA polymerase is homologous to those encoded by bacteriophages T3 and T7. Cell 51: 89–99. 10.1016/0092-8674(87)90013-4 3308116

[bib36] Matic S, Jiang M, Nicholls TJ, Uhler JP, Dirksen-Schwanenland C, Polosa PL, Simard M-L, Li X, Atanassov I, Rackham O, (2018) Mice lacking the mitochondrial exonuclease MGME1 accumulate mtDNA deletions without developing progeria. Nat Commun 9: 1202. 10.1038/s41467-018-03552-x 29572490 PMC5865154

[bib37] Milenkovic D, Matic S, Kühl I, Ruzzenente B, Freyer C, Jemt E, Park CB, Falkenberg M, Larsson N-G (2013) TWINKLE is an essential mitochondrial helicase required for synthesis of nascent D-loop strands and complete mtDNA replication. Hum Mol Genet 22: 1983–1993. 10.1093/hmg/ddt051 23393161 PMC3633371

[bib73] Minczuk M, He J, Duch AM, Ettema TJ, Chlebowski A, Dzionek K, Nijtmans LGJ, Huynen MA, Holt IJ (2011) TEFM (c17orf42) is necessary for transcription of human mtDNA. Nucleic Acids Res 39. 10.1093/nar/gkq1224 PMC3105396 21278163

[bib38] Miranda M, Bonekamp NA, Kühl I (2022) Starting the engine of the powerhouse: Mitochondrial transcription and beyond. Biol Chem 403: 779–805. 10.1515/hsz-2021-0416 35355496

[bib39] Misic J, Milenkovic D, Al-Behadili A, Xie X, Jiang M, Jiang S, Filograna R, Koolmeister C, Siira SJ, Jenninger L, (2022) Mammalian RNase H1 directs RNA primer formation for mtDNA replication initiation and is also necessary for mtDNA replication completion. Nucleic Acids Res 50: 8749–8766. 10.1093/nar/gkac661 35947649 PMC9410905

[bib40] Montoya J, Christianson T, Levens D, Rabinowitz M, Attardi G (1982) Identification of initiation sites for heavy-strand and light-strand transcription in human mitochondrial DNA. Proc Natl Acad Sci U S A 79: 7195–7199. 10.1073/pnas.79.23.7195 6185947 PMC347305

[bib41] Morgenstern M, Peikert CD, Lübbert P, Suppanz I, Klemm C, Alka O, Steiert C, Naumenko N, Schendzielorz A, Melchionda L, (2021) Quantitative high-confidence human mitochondrial proteome and its dynamics in cellular context. Cell Metab 33: 2464–2483.e18. 10.1016/j.cmet.2021.11.001 34800366 PMC8664129

[bib42] Morozov YI, Parshin AV, Agaronyan K, Cheung ACM, Anikin M, Cramer P, Temiakov D (2015) A model for transcription initiation in human mitochondria. Nucleic Acids Res 43: 3726–3735. 10.1093/nar/gkv235 25800739 PMC4402542

[bib43] Ojala D, Montoya J, Attardi G (1981) tRNA punctuation model of RNA processing in human mitochondria. Nature 290: 470–474. 10.1038/290470a0 7219536

[bib44] Oláhová M, Peter B, Szilagyi Z, Diaz-Maldonado H, Singh M, Sommerville EW, Blakely EL, Collier JJ, Hoberg E, Stránecký V, (2021) POLRMT mutations impair mitochondrial transcription causing neurological disease. Nat Commun 12: 1135. 10.1038/s41467-021-21279-0 33602924 PMC7893070

[bib45] Park CB, Asin-Cayuela J, Cámara Y, Shi Y, Pellegrini M, Gaspari M, Wibom R, Hultenby K, Erdjument-Bromage H, Tempst P, (2007) MTERF3 is a negative regulator of mammalian mtDNA transcription. Cell 130: 273–285. 10.1016/j.cell.2007.05.046 17662942

[bib69] Pertea M, Kim D, Pertea GM, Leek JT, Salzberg SL (2016) Trancript-level expression analysis of RNA-seq experiments with HISAT, StringTie and Ballgown. Nat Protoc 11: 1650–1667. 10.1038/nprot.2016.095 27560171 PMC5032908

[bib74] Posse V, Shahzad S, Falkenberg M, Hällberg BM, Gustafsson CM (2015) TEFM is a potent stimulator of mitochondrial transcription elongation in vitro. Nucleic Acids Res 43. 10.1093/nar/gkv105 PMC4357710 25690892

[bib72] R Development Core Team (2010) R: A Language and Environment for Statistical Computing. Vienna, Austria: R Foundation for Statistical Computing. Available at: http://www.R-project.org.

[bib46] Rackham O, Busch JD, Matic S, Siira SJ, Kuznetsova I, Atanassov I, Ermer JA, Shearwood A-MJ, Richman TR, Stewart JB, (2016) Hierarchical RNA processing is required for mitochondrial ribosome assembly. Cell Rep 16: 1874–1890. 10.1016/j.celrep.2016.07.031 27498866

[bib71] Rappsilber J, Ishihama Y, Mann M (2003) Stop and go extraction tips for matrix-assisted laser desorption/ionization, nanoelectrospray, and LC/MS sample pretreatment in proteomics. Anal Chem 75: 663–670. 10.1021/ac026117i 12585499

[bib47] Rath S, Sharma R, Gupta R, Ast T, Chan C, Durham TJ, Goodman RP, Grabarek Z, Haas ME, Hung WHW, (2021) MitoCarta3.0: An updated mitochondrial proteome now with sub-organelle localization and pathway annotations. Nucleic Acids Res 49: D1541–D1547. 10.1093/nar/gkaa1011 33174596 PMC7778944

[bib48] Ringel R, Sologub M, Morozov YI, Litonin D, Cramer P, Temiakov D (2011) Structure of human mitochondrial RNA polymerase. Nature 478: 269–273. 10.1038/nature10435 21947009

[bib51] Rubalcava-Gracia D, García-Villegas R, Larsson N-G (2023) No role for nuclear transcription regulators in mammalian mitochondria? Mol Cell 83: 832–842. 10.1016/j.molcel.2022.09.010 36182692

[bib52] Rubalcava-Gracia D, Bubb K, Levander F, Burr SP, August AV, Chinnery PF, Koolmeister C, Larsson N-G (2024) LRPPRC and SLIRP synergize to maintain sufficient and orderly mammalian mitochondrial translation. Nucleic Acids Res 52: 11266–11282. 10.1093/nar/gkae662 39087558 PMC11472161

[bib53] Ruzzenente B, Metodiev MD, Wredenberg A, Bratic A, Park CB, Cámara Y, Milenkovic D, Zickermann V, Wibom R, Hultenby K, (2012) LRPPRC is necessary for polyadenylation and coordination of translation of mitochondrial mRNAs: LRPPRC regulates mitochondrial translation. EMBO J 31: 443–456. 10.1038/emboj.2011.392 22045337 PMC3261557

[bib54] Sarfallah A, Zamudio-Ochoa A, Anikin M, Temiakov D (2021) Mechanism of transcription initiation and primer generation at the mitochondrial replication origin OriL. EMBO J 40: e107988. 10.15252/embj.2021107988 34423452 PMC8488568

[bib55] Sasarman F, Brunel-Guitton C, Antonicka H, Wai T, Shoubridge EA, LSFC Consortium (2010) LRPPRC and SLIRP interact in a ribonucleoprotein complex that regulates posttranscriptional gene expression in mitochondria. Mol Biol Cell 21: 1315–1323. 10.1091/mbc.e10-01-0047 20200222 PMC2854090

[bib56] Schwinghammer K, Cheung ACM, Morozov YI, Agaronyan K, Temiakov D, Cramer P (2013) Structure of human mitochondrial RNA polymerase elongation complex. Nat Struct Mol Biol 20: 1298–1303. 10.1038/nsmb.2683 24096365 PMC4321815

[bib57] Siira SJ, Rossetti G, Richman TR, Perks K, Ermer JA, Kuznetsova I, Hughes L, Shearwood AMJ, Viola HM, Hool LC, (2018) Concerted regulation of mitochondrial and nuclear non-coding RNAs by a dual-targeted RNase Z. EMBO Rep 19: e46198. 10.15252/embr.201846198 30126926 PMC6172459

[bib58] Silva Ramos E, Motori E, Brüser C, Kühl I, Yeroslaviz A, Ruzzenente B, Kauppila JHK, Busch JD, Hultenby K, Habermann BH, (2019) Mitochondrial fusion is required for regulation of mitochondrial DNA replication. PLoS Genet 15: e1008085. 10.1371/journal.pgen.1008085 31170154 PMC6553695

[bib77] Sotgia F, Whitaker-Menesez D, Matrinez-Outschoorn UE, Salem AF, Tsirigos A, Lamb R, Sneddon S, Hulit J, Howell A, Lisanti MP (2012) Mitochondria “fuel” breast cancer metabolism: Fifteen markers of mitochondrial biogenesis label epithelial cancer cells, but are excluded from adjacent stromal cells. Cell Cycle 11: 4390–4401. 10.4161/cc.22777 23172368 PMC3552922

[bib59] Surovtseva YV, Shadel GeraldS (2013) Transcription-independent role for human mitochondrial RNA polymerase in mitochondrial ribosome biogenesis. Nucleic Acids Res 41: 2479–2488. 10.1093/nar/gks1447 23303773 PMC3575816

[bib60] Tan BG, Mutti CD, Shi Y, Xie X, Zhu X, Silva-Pinheiro P, Menger KE, Díaz-Maldonado H, Wei W, Nicholls TJ, (2022) The human mitochondrial genome contains a second light strand promoter. Mol Cell 82: 3646–3660.e9. 10.1016/j.molcel.2022.08.011 36044900 PMC7619432

[bib61] Terzioglu M, Ruzzenente B, Harmel J, Mourier A, Jemt E, López MD, Kukat C, Stewart JB, Wibom R, Meharg C, (2013) MTERF1 binds mtDNA to prevent transcriptional interference at the light-strand promoter but is dispensable for rRNA gene transcription regulation. Cell Metab 17: 618–626. 10.1016/j.cmet.2013.03.006 23562081

[bib62] Tyanova S, Temu T, Sinitcyn P, Carlson A, Hein MY, Geiger T, Mann M, Cox J (2016) The Perseus computational platform for comprehensive analysis of (prote)omics data. Nat Methods 13: 731–740. 10.1038/nmeth.3901 27348712

[bib63] Wang H, Liu Y, Lu X, Wu Y, Gu W, Yin G (2024) Targeting POLRMT by IMT1 inhibits colorectal cancer cell growth. Cell Death Dis 15: 643. 10.1038/s41419-024-07023-8 39227564 PMC11372113

[bib64] Wanrooij PH, Uhler JP, Shi Y, Westerlund F, Falkenberg M, Gustafsson CM (2012) A hybrid G-quadruplex structure formed between RNA and DNA explains the extraordinary stability of the mitochondrial R-loop. Nucleic Acids Res 40: 10334–10344. 10.1093/nar/gks802 22965135 PMC3488243

[bib76] Wanrooij S, Miralles Fusté J, Farge G, Shi Y, Gustafsson CM, Falkenberg M (2008) Human mitochondrial RNA polymerase primes lagging-strand DNA synthesis. PNAS 105: 11122–11127. 10.1073/pnas.0805399105 18685103 PMC2516254

[bib65] Whitehall JC, Greaves LC (2020) Aberrant mitochondrial function in ageing and cancer. Biogerontology 21: 445–459. 10.1007/s10522-019-09853-y 31802313 PMC7347693

[bib66] Zhou T, Sang YH, Cai S, Xu C, Shi MH (2021) The requirement of mitochondrial RNA polymerase for non-small cell lung cancer cell growth. Cell Death Dis 12: 751. 10.1038/s41419-021-04039-2 34326320 PMC8322058

[bib67] Zhu X, Xie X, Das H, Tan BG, Shi Y, Al-Behadili A, Peter B, Motori E, Valenzuela S, Posse V, (2022) Non-coding 7S RNA inhibits transcription via mitochondrial RNA polymerase dimerization. Cell 185: 2309–2323.e24. 10.1016/j.cell.2022.05.006 35662414

