# Supplementary figures and images for: POLRMT overexpression increases mtDNA transcription without affecting steady-state mRNA levels
Source: Life Sci Alliance. 2025 Oct 17;8(12):e202302563. doi: 10.26508/lsa.202302563 (PMC12534795; doi:10.26508/lsa.202302563)

FIGURE 1

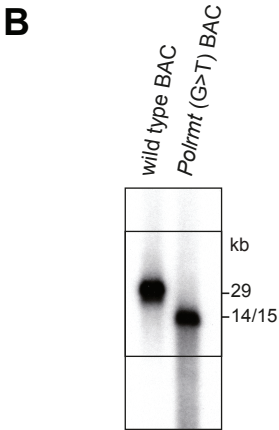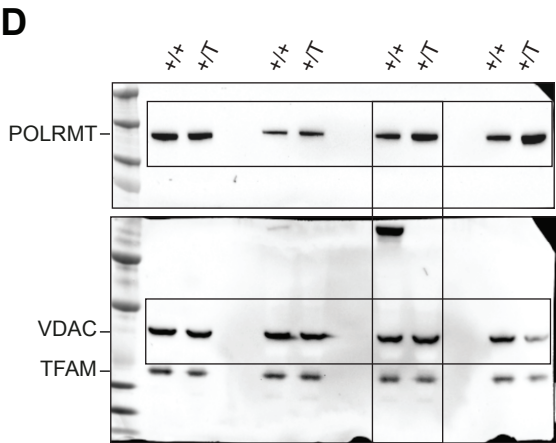

Supplement: Supplementary file 2 [file LSA-2023-02563_SdataF1.2.pdf]

FIGURE 3

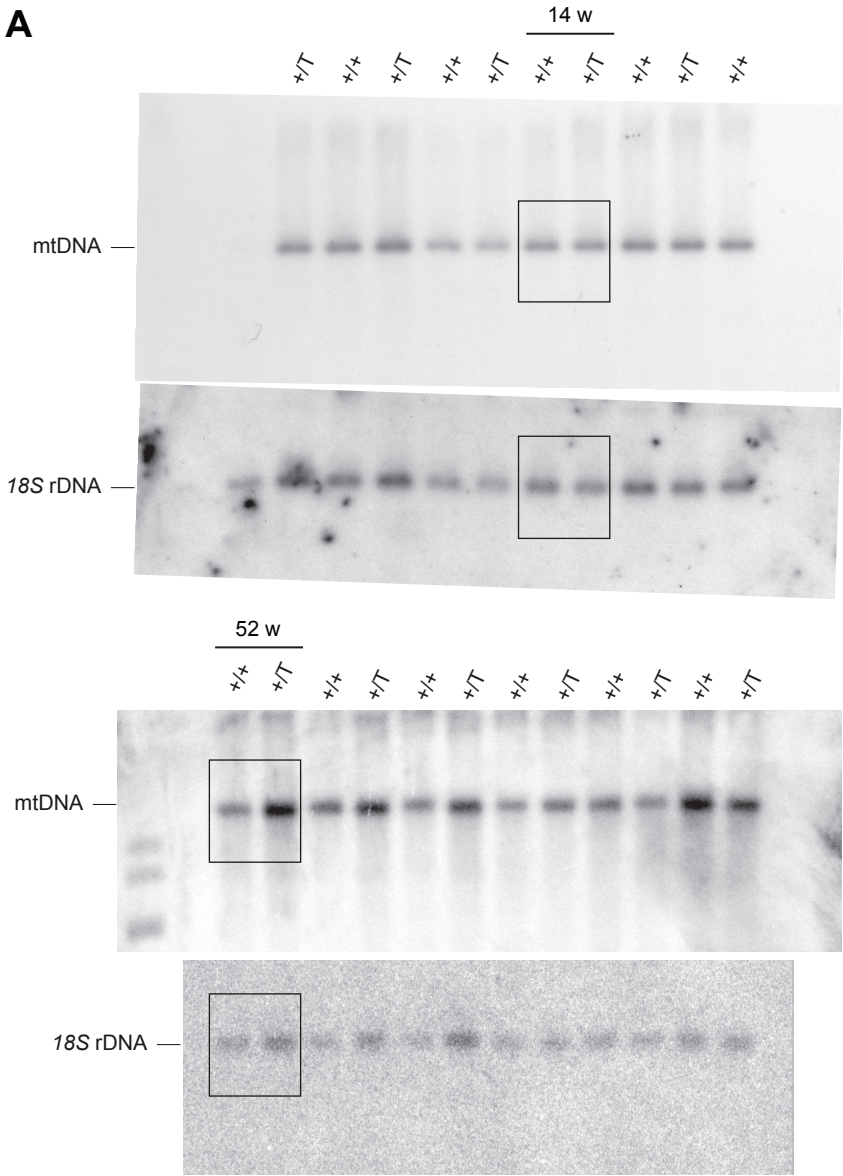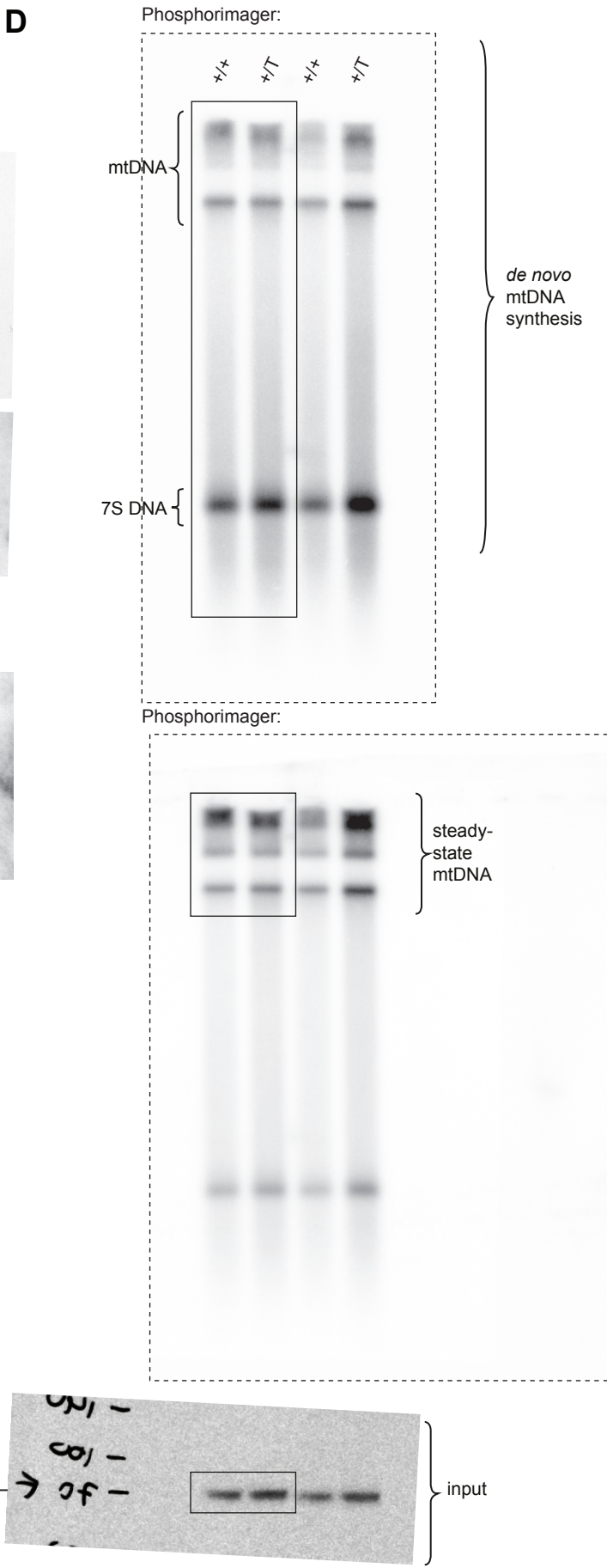

**FIGURE 3**

**F**

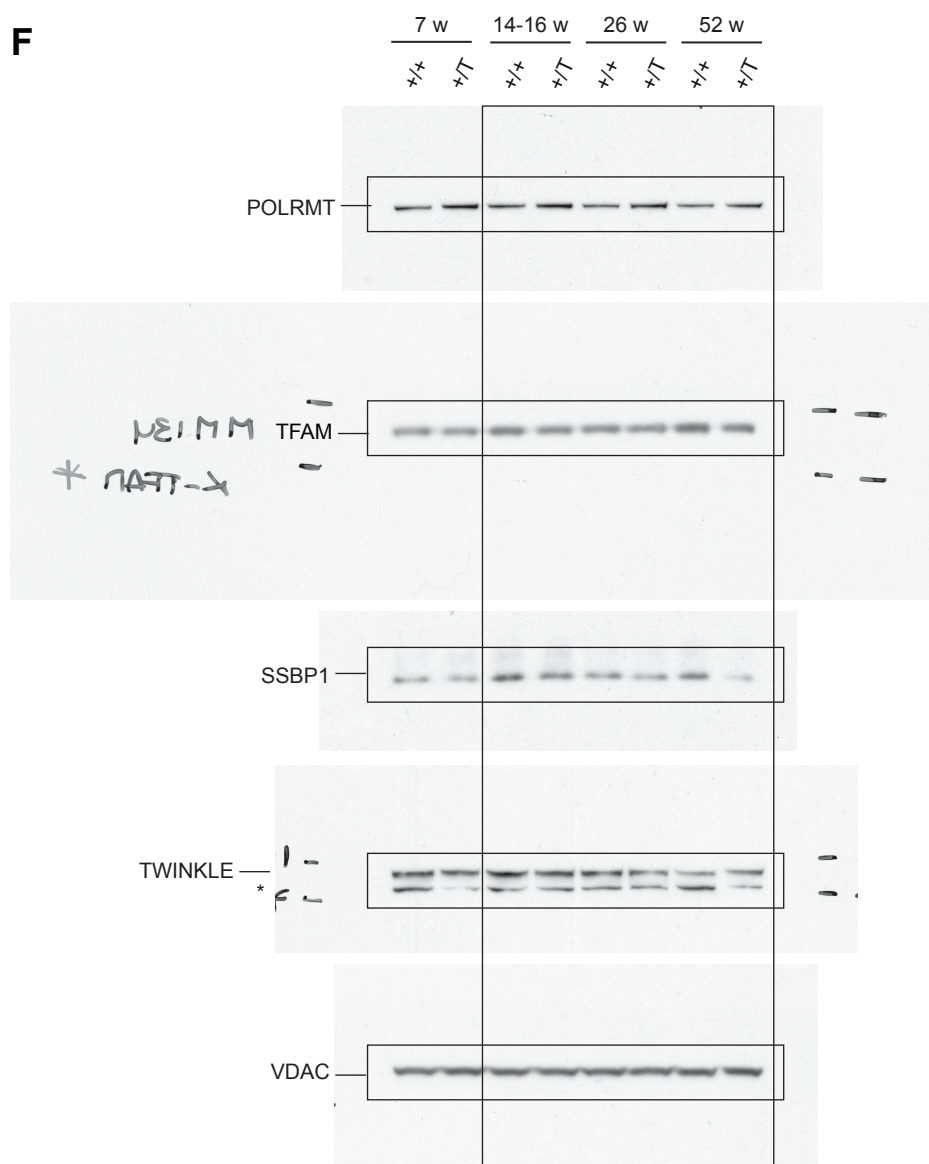

Supplement: Supplementary file 4 [file LSA-2023-02563_SdataF3.1.pdf]

**FIGURE 4**

**A**

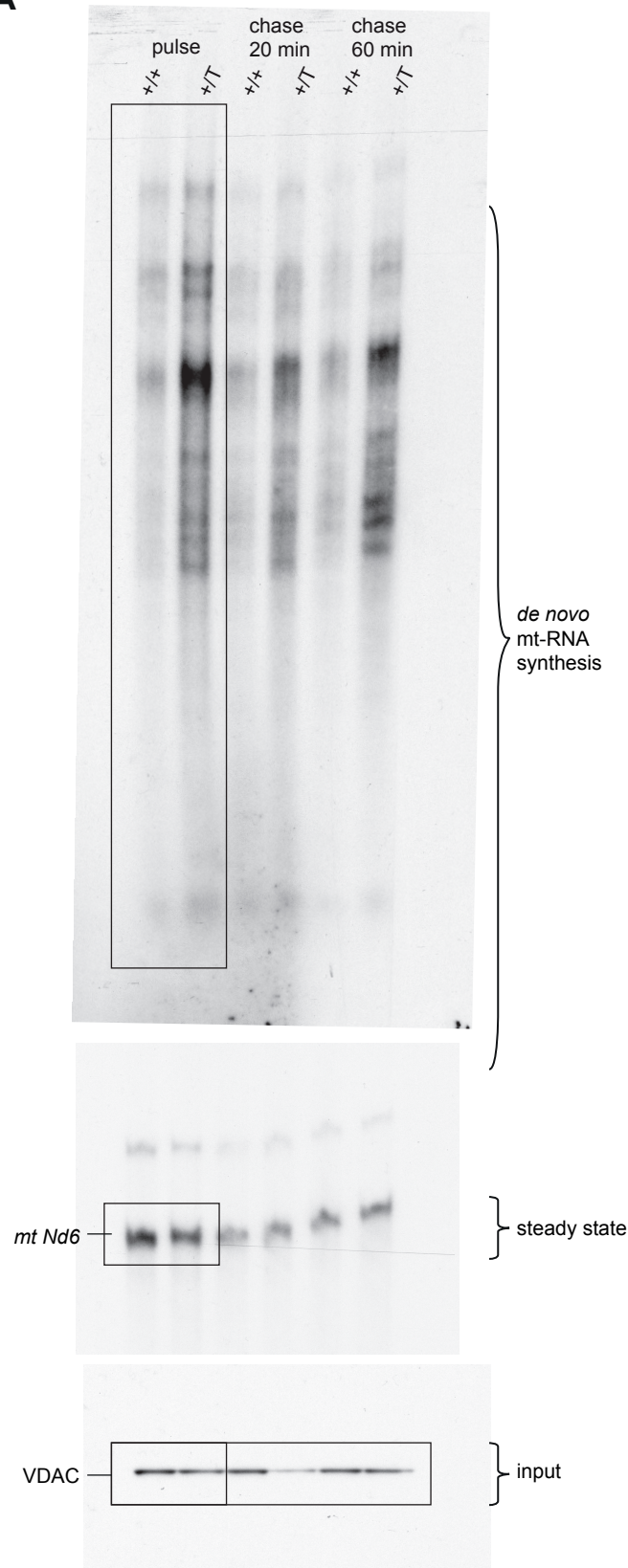

FIGURE 4

D

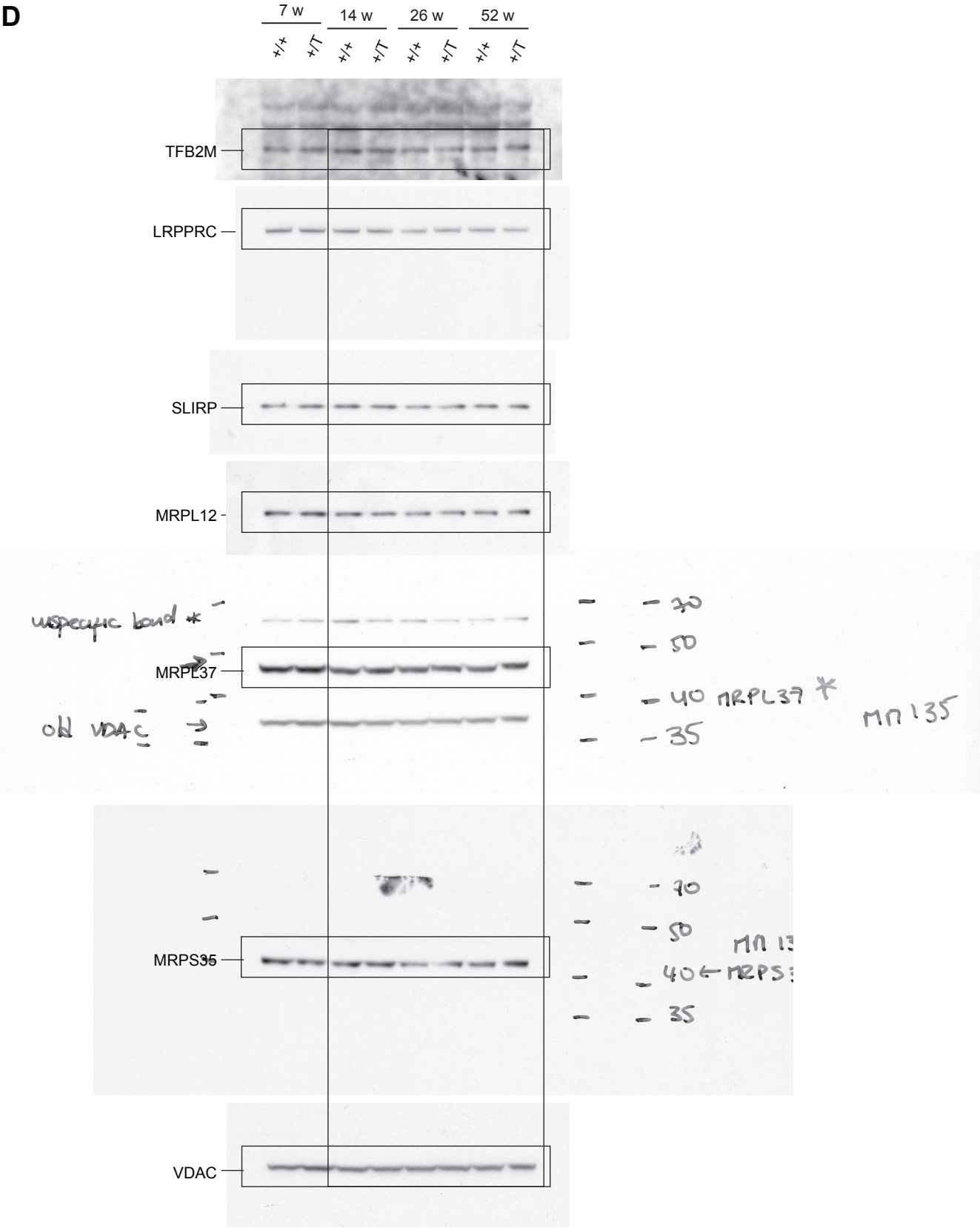

FIGURE 4

D

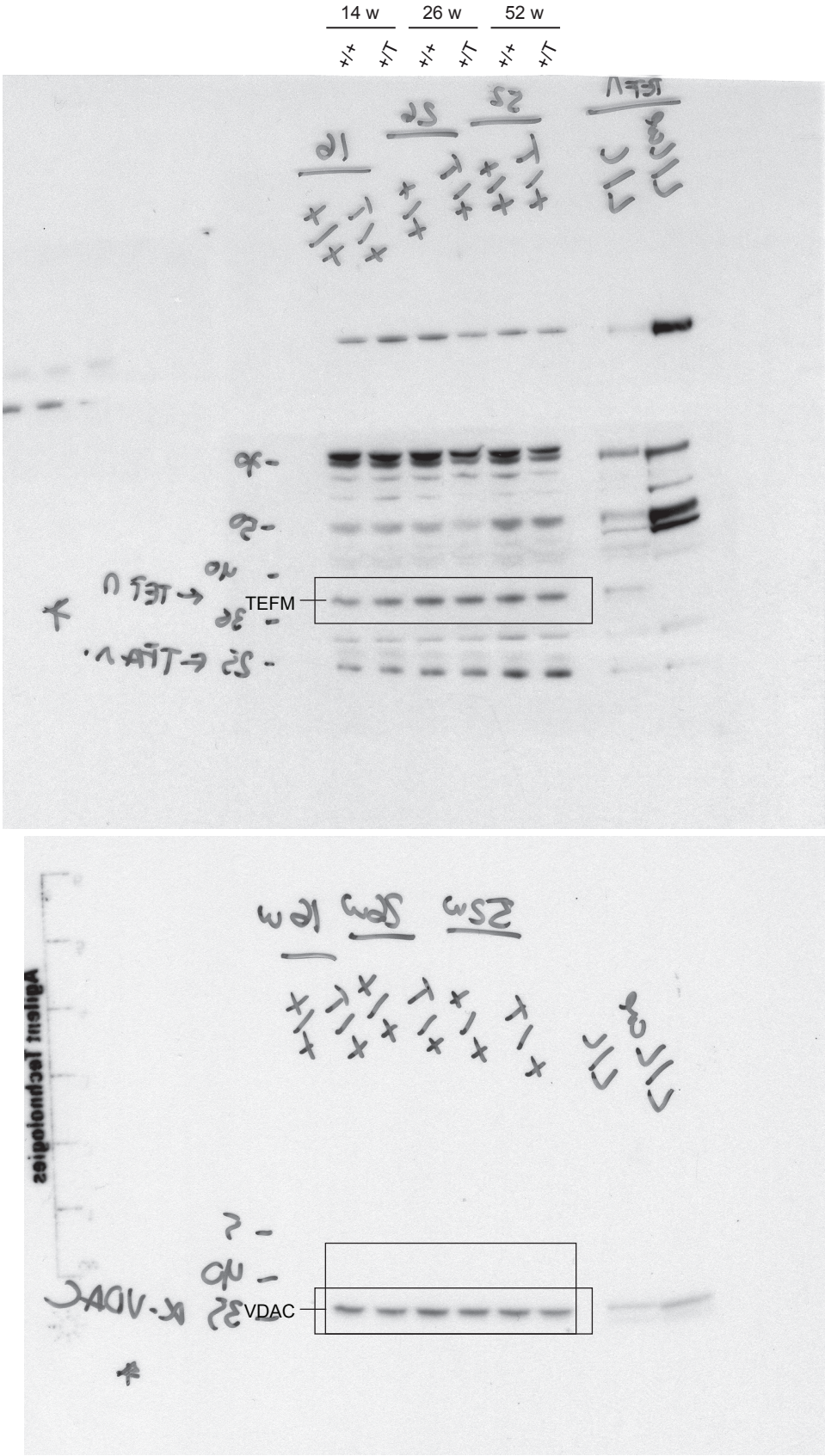

D

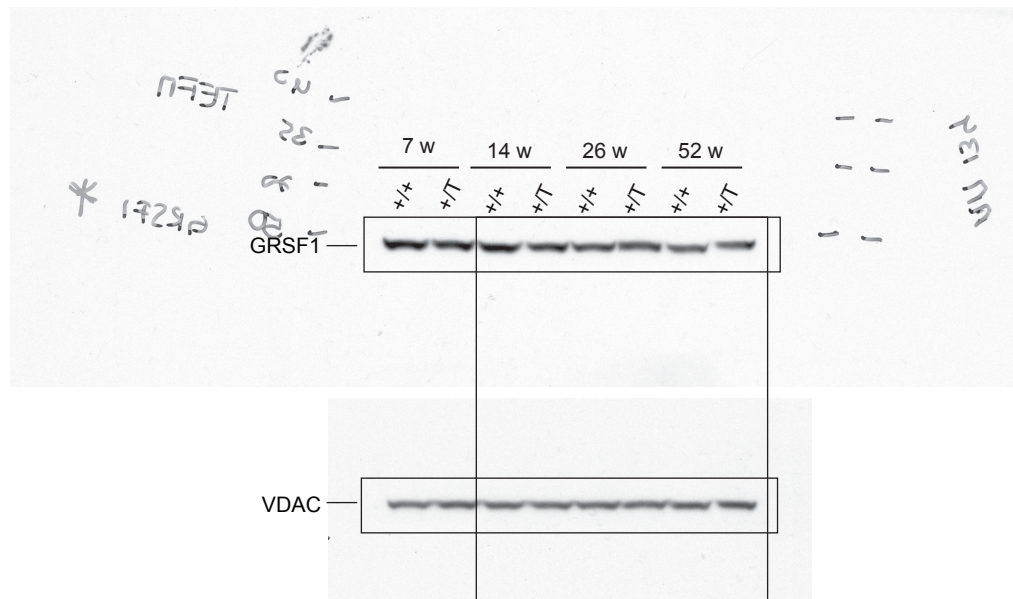

Supplement: Supplementary file 6 [file LSA-2023-02563_SdataF4.1.pdf]

**FIGURE 5**  
**C - 14-16 w**

Phosphorimager:

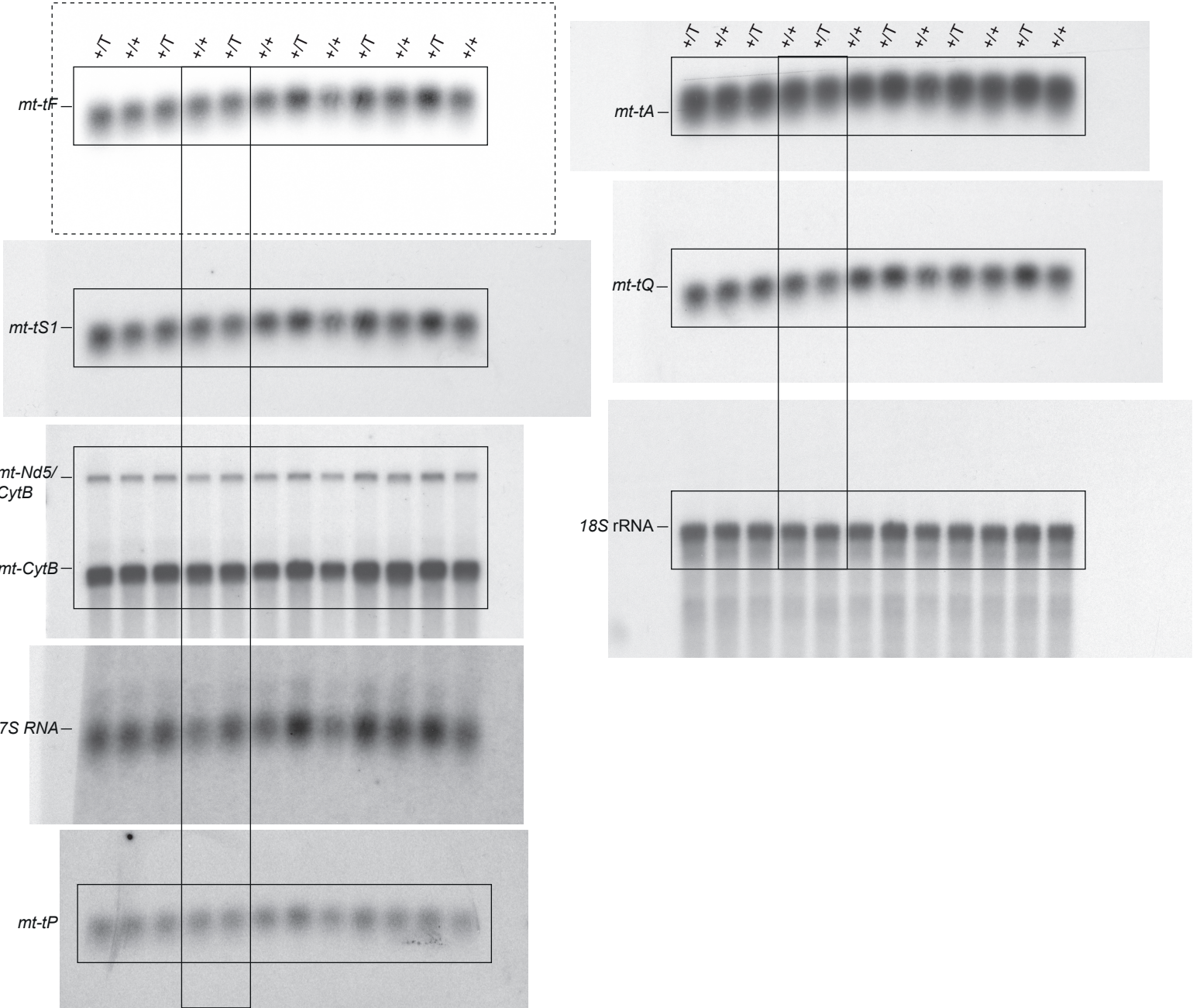

**FIGURE 5**  
**C - 52-58w**

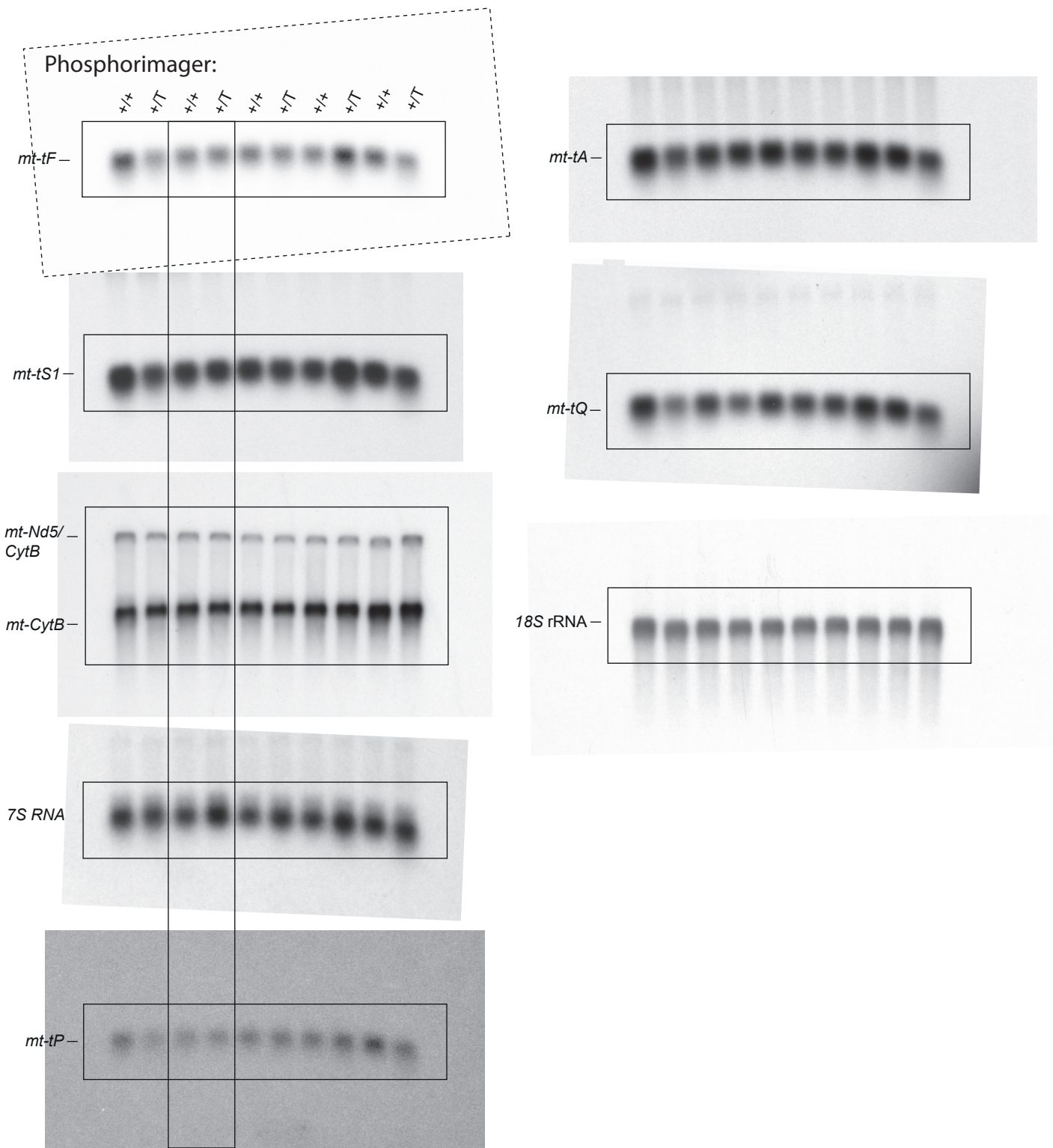

**FIGURE 5**

**E**

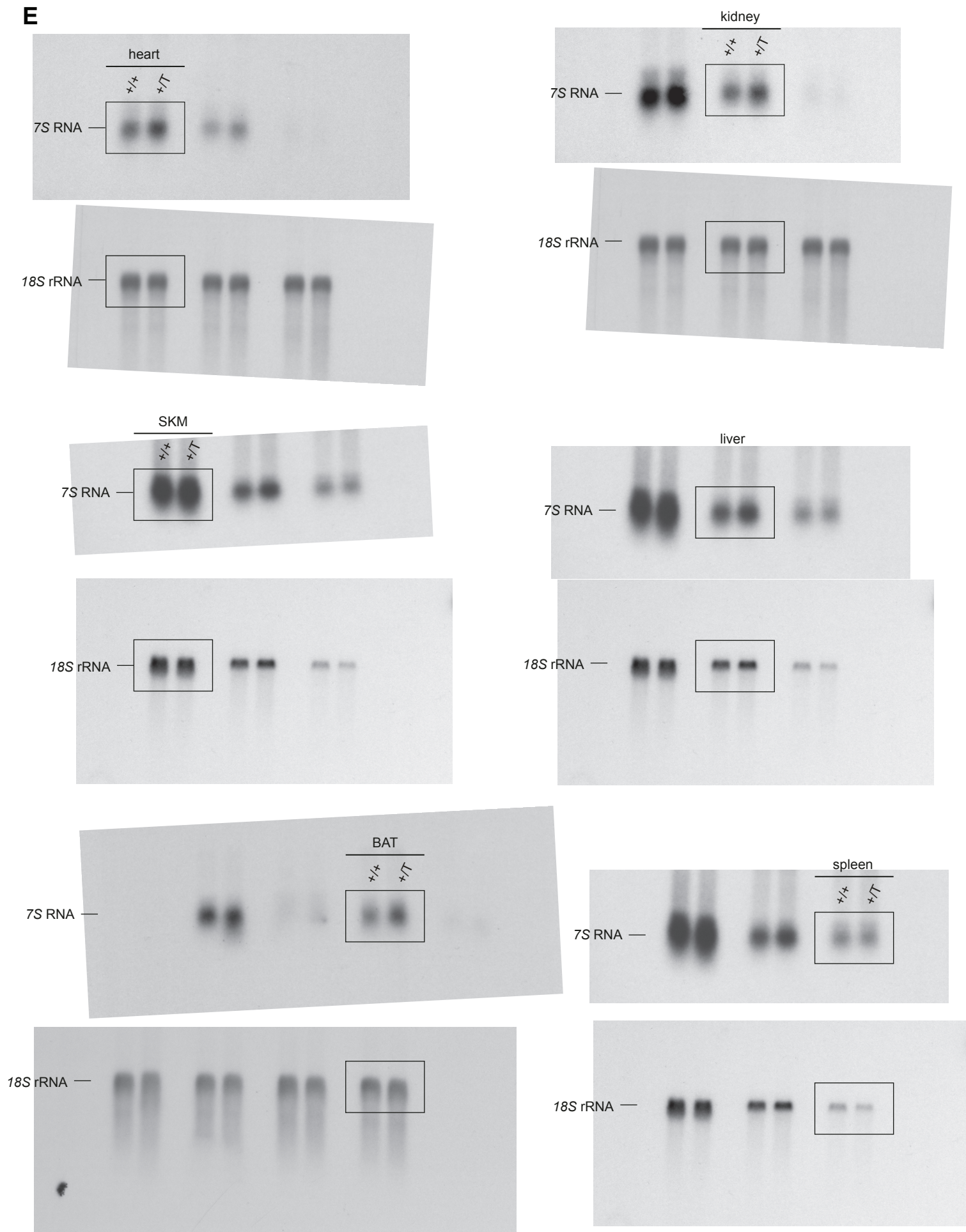

Supplement: Supplementary file 8 [file LSA-2023-02563_SdataF5.1.pdf]

FIGURE 6  
A

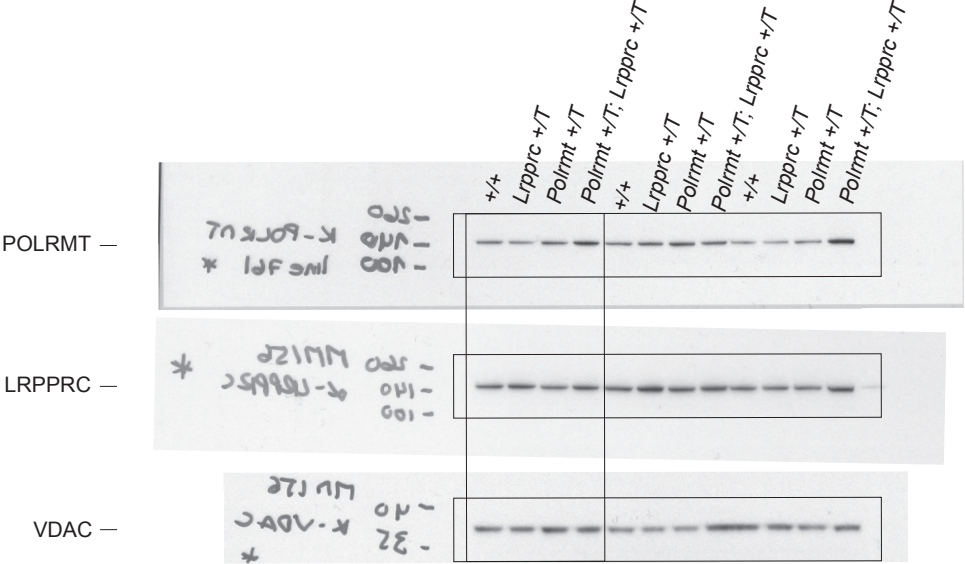

C

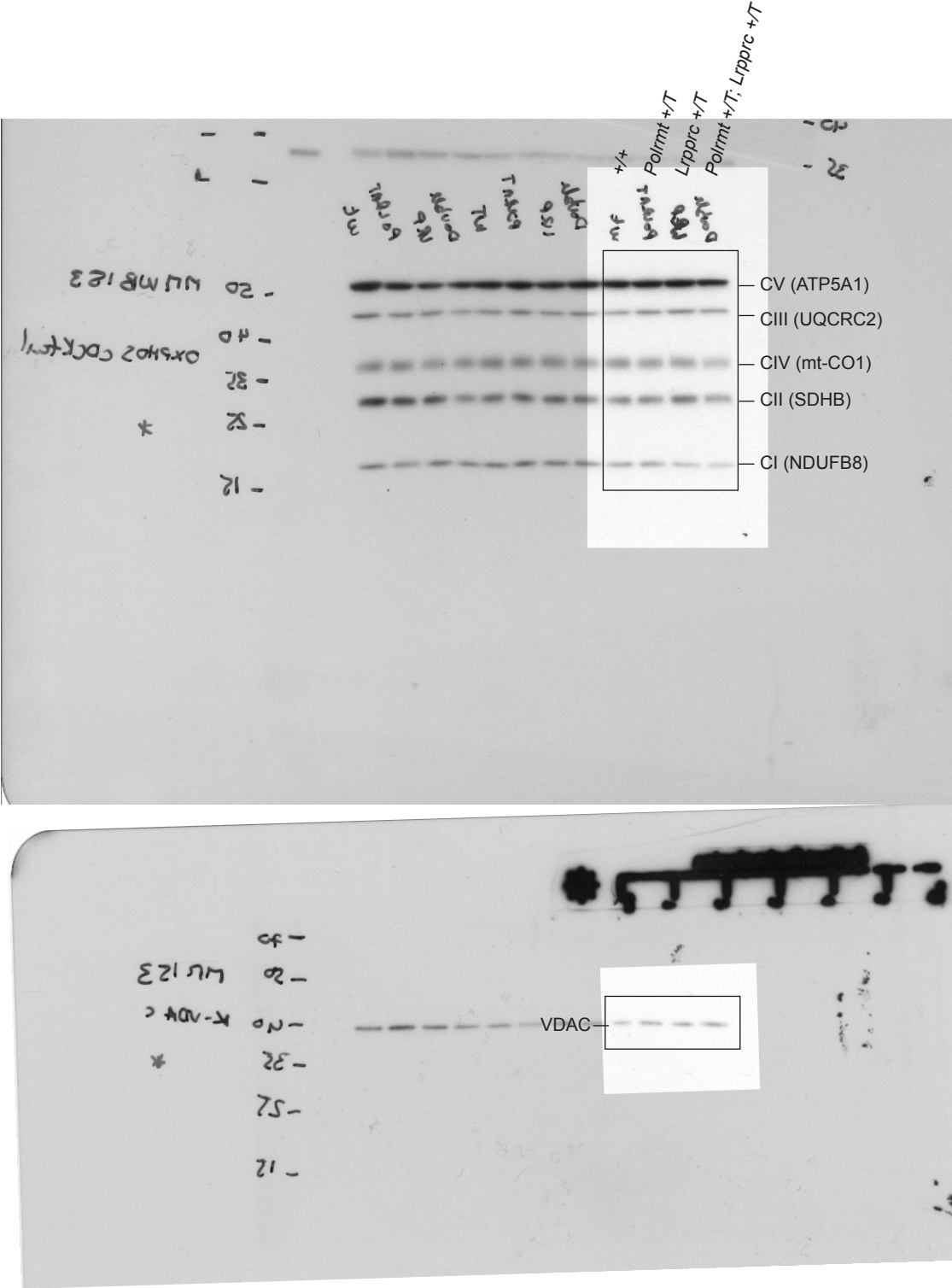

Supplement: Supplementary file 10 [file LSA-2023-02563_SdataF6.1.pdf]
