# Supplementary material for: POLRMT overexpression increases mtDNA transcription without affecting steady-state mRNA levels
Source: Life Sci Alliance. 2025 Oct 17;8(12):e202302563. doi: 10.26508/lsa.202302563 (PMC12534795; doi:10.26508/lsa.202302563)
Supplement: Reviewer comments [file LSA-2023-02563_review_history.pdf]

Life Science Alliance

POLRMT overexpression increases mtDNA transcription without affecting steady-state mRNA levels

Maria Miranda, Andrea Mesaros, Nathalie Scrima, Louise Pérard, Irina Kuznetsova, Martin Purrio, Ilian Atanassov, Aleksandra Filipovska, Arnaud Mourier, Nils-Göran Larsson and Inge Kühl

DOI: https://doi.org/10.26508/lsa.202302563

Corresponding author(s): Dr. Inge Kühl (Institute of Integrative Biology of the Cell)

Review Timeline:

Submission Date:	2023-12-29
Editorial Decision:	2024-02-12
Revision Received:	2025-07-26
Editorial Decision:	2025-09-04
Revision Received:	2025-09-22
Accepted:	2025-09-24

Scientific Editor: Tim Fessenden

Transaction Report:

(Note: With the exception of the correction of typographical or spelling errors that could be a source of ambiguity, letters and reports are not edited. The original formatting of letters and referee reports may not be reflected in this compilation.)

No full Peer Review Process File is available with this article, as the authors have chosen not to make the review process public in this case.

February 12, 2024

Re: Life Science Alliance manuscript #LSA-2023-02563

Prof. Inge Kühl
Institute of Integrative Biology of the Cell
Avenue de la Terrasse 1
Gif-sur-Yvette 91190
France

Dear Dr. Kühl,

Thank you for submitting your manuscript entitled "Increased mitochondrial transcription initiation does not promote oxidative phosphorylation". The manuscript has been evaluated by expert reviewers, whose reports are appended below. Unfortunately, after an assessment of the reviewer feedback, our editorial decision is against publication in Life Science Alliance.

Although your manuscript is intriguing, I feel that the points raised by the reviewers are more substantial than can be addressed in a typical revision period. If you wish to expedite publication of the current data, it may be best to pursue publication at another journal.

Given the interest in the topic, I would be open to re-submission to Life Science Alliance of a significantly revised and extended manuscript that fully addresses the reviewers' concerns and is subject to further peer review. If you would like to resubmit this work to Life Science Alliance, you may submit an appeal directly through our manuscript submission system. Please note that priority and novelty would be reassessed at re-submission.

Regardless of how you choose to proceed, we hope that the comments below will prove constructive as your work progresses.

Thank you for thinking of Life Science Alliance as an appropriate place to publish your work.

Sincerely,

Eric Sawey, PhD
Executive Editor
Life Science Alliance
http://www.lsajournal.org

September 4, 2025

RE: Life Science Alliance Manuscript #LSA-2023-02563R-A

Dr. Inge Kühl
Institute of Integrative Biology of the Cell
Cell Biology
Avenue de la Terrasse 1
Gif-sur-Yvette 91190
France

Dear Dr. Kühl,

Thank you for submitting your revised manuscript entitled "Activation of mtDNA transcription by POLRMT overexpression enhances stress-induced exercise capacity". This work was returned to the original reviewers whose reports are below. Overall, given the several improvements in this work and the clear support conveyed by Reviewers, we would be happy to publish your paper in Life Science Alliance pending the above changes and final revisions necessary to meet our formatting guidelines.

Along with points mentioned below, please tend to the following:
-Please upload your main and supplementary figures as single files.
-Please add the X and Bluesky handles of your host institute/organization, as well as your own and/or one of the authors, in our system.
-Please be sure that all authors are mentioned in the author contribution section in the manuscript text.
-Please use the [10 author names, et al.] format in your references (i.e., limit the author names to the first 10).
-We encourage you to revise the figure legends for figures 1 and S1 such that the figure panels are introduced in alphabetical order.
-Please remove the reference to panel C from the legend for Figure S7.
-Please add callouts for Figures S5B; S6A-B and S7 to your main manuscript text.
-Please add a Data Availability section to your manuscript, in which we request you provide a link to the mass spectrometry data at a public repository.

If you are planning a press release on your work, please inform us immediately to allow informing our production team and scheduling a release date.

LSA now encourages authors to provide a 30-60 second video where the study is briefly explained. We will use these videos on social media to promote the published paper and the presenting author (for examples, see https://docs.google.com/document/d/1-UWCfbE4pGcDdcgzcmiuJl2XMBJnxKYeqRvLLrLSo8s/edit?usp=sharing). Corresponding or first-authors are welcome to submit the video. Please submit only one video per manuscript. The video can be emailed to contact@life-science-alliance.org

To upload the final version of your manuscript, please log in to your account: https://lsa.msubmit.net/cgi-bin/main.plex
You will be guided to complete the submission of your revised manuscript and to fill in all necessary information. Please get in touch in case you do not know or remember your login name.

To avoid unnecessary delays in the acceptance and publication of your paper, please read the following information carefully.

A. FINAL FILES:

These items are required for acceptance.

-- An editable version of the final text (.DOC or .DOCX) is needed for copyediting (no PDFs).

-- High-resolution figure, supplementary figure and video files uploaded as individual files: See our detailed guidelines for preparing your production-ready images, https://www.life-science-alliance.org/authors

-- Summary blurb (enter in submission system): A short text summarizing in a single sentence the study (max. 200 characters including spaces). This text is used in conjunction with the titles of papers, hence should be informative and complementary to the title. It should describe the context and significance of the findings for a general readership; it should be written in the present tense and refer to the work in the third person. Author names should not be mentioned.

B. MANUSCRIPT ORGANIZATION AND FORMATTING:

Full guidelines are available on our Instructions for Authors page, https://www.life-science-alliance.org/authors

We encourage our authors to provide original source data, particularly uncropped/-processed electrophoretic blots and spreadsheets for the main figures of the manuscript. If you would like to add source data, we would welcome one PDF/Excel-file per figure for this information. These files will be linked as supplementary "Source Data" files.

Submission of a paper that does not conform to Life Science Alliance guidelines will delay the acceptance of your manuscript.

It is Life Science Alliance policy that if requested, original data images must be made available to the editors. Failure to provide original images upon request will result in unavoidable delays in publication. Please ensure that you have access to all original data images prior to final submission.

The license to publish form must be signed before your manuscript can be sent to production. A link to the license to publish form will be available to the corresponding author only. Please take a moment to check your funder requirements.

Reviews, decision letters, and point-by-point responses associated with peer-review at Life Science Alliance will be published, alongside the manuscript. If you do want to opt out of having the reviewer reports and your point-by-point responses displayed, please let us know immediately.

Thank you for your attention to these final processing requirements. Please revise and format the manuscript and upload materials as soon as you are able.

Thank you for this interesting contribution, we look forward to publishing your paper in Life Science Alliance.

Sincerely,

Tim Fessenden
Scientific Editor
Life Science Alliance

September 24, 2025

RE: Life Science Alliance Manuscript #LSA-2023-02563RR

Dr. Inge Kühl
Institute of Integrative Biology of the Cell
Cell Biology
Avenue de la Terrasse 1
Gif-sur-Yvette 91190
France

Dear Dr. Kühl,

Thank you for submitting your Research Article entitled "POLRMT overexpression increases mtDNA transcription without affecting steady-state mRNA levels". It is a pleasure to let you know that your manuscript is now accepted for publication in Life Science Alliance. Congratulations on this interesting work.

The final published version of your manuscript will be deposited by us to PubMed Central upon publication.

Your manuscript will now progress through copyediting and proofing. It is journal policy that authors provide original data upon request.

IMPORTANT: If you will be unreachable at any time, please provide us with the email address of an alternate author. Failure to respond to routine queries may lead to unavoidable delays in publication.

Scheduling details will be available from our production department. You will receive proofs shortly before the publication date. Only essential corrections can be made at the proof stage so if there are any minor final changes you wish to make to the manuscript, please let the journal office know now.

DISTRIBUTION OF MATERIALS:
Authors are required to distribute freely any materials used in experiments published in Life Science Alliance. Authors are encouraged to deposit materials used in their studies to the appropriate repositories for distribution to researchers.

You can contact the journal office with any questions, contact@life-science-alliance.org

Again, congratulations on a very nice paper. I hope you found the review process to be constructive and are pleased with how the manuscript was handled editorially. We look forward to future exciting submissions from your lab.

Sincerely,

Tim Fessenden
Scientific Editor
Life Science Alliance
